# Identification of priority shorebird conservation areas in the Caribbean

**DOI:** 10.7717/peerj.9831

**Published:** 2020-09-08

**Authors:** Jessica R. Cañizares, J. Michael Reed

**Affiliations:** Biology Department, Tufts University, Medford, MA, United States of America

**Keywords:** IBA, International Shorebird Survey, Caribbean Waterbird Census, Ramsar, WHSRN, Wetland, Important Bird and Biodiversity Area, Western Hemisphere Shorebird Reserve Network, eBird

## Abstract

Despite being geographically central to the Atlantic Americas Flyway for migratory birds, the Caribbean is often overlooked or underappreciated when addressing the conservation of North American shorebirds. To our knowledge, this is the first Caribbean-wide assessment of shorebird use in the region. We analyzed 211,013 shorebird species observations in the insular Caribbean from 2010–2019, representing 78,794 eBird checklists and cumulative total of 2.1 million shorebirds of 45 species. We conclude that priority areas for shorebird conservation include Humedal Sur de Pinar del Río (Humedal Sur de Los Palacios) in Cuba, and Monte Cristi in the Dominican Republic as they each likely support more than 20,000 shorebirds annually, and they host large abundances of geographic populations for particular taxa. Specifically, the former site hosts >10% of Short-billed Dowitchers (*Limnodromus griseus griseus/hendersoni*), and >1% of Black-bellied Plovers (*Pluvialis squatarola cynosurae*) and Wilson’s Plovers (*Charadrius wilsonia wilsonia*), while the latter site supports large numbers of Black-necked Stilts (*Himantopus mexicanus*)*.* We also identified at least 15 additional sites that likely cross the 1% population threshold for one or more shorebird taxa. These sites may qualify for special international designations such as Important Bird and Biodiversity Areas or as part of the Western Hemisphere Shorebird Reserve Network; 11 of the 17 sites we identified do not hold either of these titles. Data on subspecific or geographic distributions of three species, Snowy Plover (*C. nivosus*), Black-necked Stilt, and Killdeer (*C. vociferous*), are insufficient to reveal if the sites with the highest abundances were mostly comprised of Caribbean populations or migrants, but the limited information suggests that they also likely exceed 1% thresholds on several islands. Based on our results, we recommend more extensive systematic surveys of shorebirds in the Caribbean, including research on turnover rates and movements between islands, as well as assimilation of shorebird survey data not yet included in the eBird portal.

## Introduction

Migratory wildlife systems around the world are at risk due to anthropogenic disturbances such as habitat loss and degradation, overexploitation, and climate change (Wilcove & Wikelski, 2008). Of the four migratory flyways for birds in North America, two, the Atlantic and Mississippi, have experienced significant declines in nocturnal biomass passage for all bird species; from 2007–2017, total migration biomass in the Atlantic Flyway decreased about 3% each year over the ten-year period (Rosenberg et al., 2019). In North America, the species that migrate the farthest distances, shorebirds, have declined by an estimated 37–60% since the 1970s, resulting in reduction in standing population size of over 17 million breeding shorebirds (North American Bird Conservation Initiative, 2016; Rosenberg et al., 2019). These declines have resulted in a number of North American taxa receiving protected status under the U.S. Endangered Species Act (three species and five populations); all numbers exclude the Eskimo Curlew, *Numenius borealis*, which is likely extinct (Elphick, Roberts & Reed, 2010), although it is still listed as endangered), the Canadian Species at Risk Act (nine taxa), and/or being listed as Near Threatened (11) or Vulnerable (one) by the International Union for the Conservation of Nature (IUCN). These North American declines (Thomas, Lanctot & Székely, 2006; Bart et al., 2007; Andres et al., 2012) are consistent with a worldwide phenomenon of shorebird declines (International Wader Study Group, 2003; Szabo et al., 2016). Despite conservation efforts, it is likely more species will be added to these lists because of climate change (Galbraith et al., 2014).

The specific mechanisms of shorebird decline for most species are not well understood, though some are known to be threatened regionally by habitat loss and harvest (Murray et al., 2014; Watts & Turrin, 2016). In addition, some aspects of shorebird life-history make them more vulnerable than other species: low reproductive rates, phenological dependencies caused by precise timing and energy requirements during migration and breeding initiation, and the tendency of some species to aggregate in high concentrations during migration and on non-breeding grounds (Myers et al., 1987; Pfister, Harrington & Lavine, 1992; Murray & Fuller, 2015). As examples of the last factor, *rufa* Red Knots (*Calidris canutus rufa*) and Semipalmated Sandpipers (*C. pusilla)* congregate in spectacular numbers, up to millions of birds for the latter species, at staging sites that are thought to be necessary to complete migration (Mawhinney, Hicklin & Boates, 1993; Niles, Sitters & Dey, 2010). Thus, loss or degradation of a single site could have enormous impacts on the success of the migratory journey and, in turn, population persistence (Brown et al., 2001; Kirby et al., 2008); this has already been reported for the *rufa* subspecies of the Red Knot (Baker et al., 2004; Escudero et al., 2012). For species depending on multiple sites throughout their annual cycle, the site with the worst conditions (e.g., higher mortality or lower carrying capacity) may drive the overall population trend regardless of conservation efforts elsewhere in a species’ range (Sutherland, 1996; Runge et al., 2014). Therefore, protecting important sites during migration and in non-breeding areas could become a higher conservation priority than protecting breeding grounds, even though species spend a disproportionately smaller amount of their annual cycle migrating (Buler & Moore, 2011; Runge et al., 2014). In a recent analysis, just 9% of 1,451 migratory birds studied occupied protected areas throughout their entire annual cycle (Runge et al., 2015).

Our interest here is in identifying Caribbean sites that are important to migratory shorebirds that are part of the Atlantic Flyway of North America, both the migrants and resident populations of the species that use the region. Several of the taxa classified as shorebirds of conservation concern by the US Shorebird Conservation Plan are found almost exclusively within the Atlantic Flyway or the Caribbean: *rufa* Red Knot, Piping Plover (*Charadrius melodus melodus*), Snowy Plover (*C. nivosus nivosus* (Gulf Coast) and *C. n. tenuirostris*) and Short-billed Dowitcher (*Limnodromus griseus griseus*) and many others use the Caribbean region as part of their annual cycle (US Shorebird Conservation Plan Partnership, 2016).

The Caribbean provides key links in the Atlantic Flyway between the continents but has been poorly studied. The Caribbean supports diverse habitats that are important for shorebirds, such as sandy beaches, lagoons, salt pans, rice fields, and mudflats. There are also 294 Important Bird and Biodiversity Areas (IBAs; http://www.datazone.birdlife.org/home; accessed 18 May 2020) and 42 Ramsar sites (wetlands of international importance) (https://rsis.ramsar.org/; accessed 18 May 2020). However, the current status of shorebird habitat across the Caribbean is poorly known, with the most recent regional wetland inventory published in the mid-1980s (Scott & Carbonell, 1986). While this review provides important information, its primary focus was waterfowl and was not comprehensive as it omitted critical wetland habitats such as the West Coast Mudflats (an IBA) in Trinidad. Moreover, for one-third of the 23 Caribbean island nations listed in their inventory, information requests went unanswered, resulting in the use of reports from the 1970s and early 1980s, or in the case of Turks and Caicos Islands, simply listing 110 wetland sites (Scott & Carbonell, 1986). Human population growth and land development have since resulted in losses of Caribbean wetlands (Bacon, 1987; Juman & Ramsewak, 2013) and the effects on Caribbean birds are largely unknown but of growing concern (Latta, 2012; Mugica et al., 2012).

Several international conservation efforts for shorebirds in the Caribbean have been initiated. These include the Western Hemisphere Shorebird Reserve Network (WHSRN), which began in 1985 (Bildstein et al., 1991; whsrn.org), and the Atlantic Flyway Shorebird Initiative (AFSI) (AFSI Business Plan, 2015; https://www.atlanticflywayshorebirds.org/). AFSI identifies the Caribbean as a priority geography and recognizes the importance of increasing shorebird conservation in the region (AFSI Business Plan, 2015). BirdLife International has classified many sites across the Caribbean that are important to shorebirds as IBAs. In addition, the International Piping Plover Census, a species-specific monitoring effort, has recently expanded their coverage outside of the United States, and found important wintering areas for the species in The Bahamas (Elliott-Smith et al., 2015).

According to their website, BirdLife International has established 21 IBAs in six Caribbean countries or territories that were triggered by shorebird species (http://www.datazone.birdlife.org/; Accessed 20 June 2020; Table S1). In 2008, BirdLife published a Caribbean inventory covering the entire IBA network in the region (283 sites at the time), with country profiles describing the inclusion criteria and conservation status of each IBA, and discussions about regional challenges such as biodiversity loss (Wege & Anadon-Irizarry, 2008). The sites important to shorebirds identified by BirdLife are a unique collection as much of the data are gathered by communicating with local experts.

In the last decade, the first two WHSRN sites in the Caribbean were designated (https://www.whsrn.org/whsrn-sites/caribbean/; accessed 18 May 2020). Cabo Rojo Salt Flats in southwest Puerto Rico was designated in 2010 because it hosts >1% of the flyway population for Snowy and Wilson’s Plovers (*C. wilsonia*). In 2018, another salt-production facility, Cargill Salt Ponds in Bonaire, was designated because it supports at least 20,000 shorebirds annually, including at least 1% of the biogeographic population of Short-billed Dowitcher (*L. g. griseus/hendersoni*) and *rufa* Red Knot. Both are listed as Sites of Regional Importance.

Our goal is to identify new priority areas for shorebird conservation in the Caribbean, and to better understand the status, abundance, and distribution of shorebirds in the region. Shorebird- or waterbird-specific protocols include the International Shorebird Survey (ISS), started in 1972 (Howe, Geissler & Harrington, 1989), and the Caribbean Waterbird Census (CWC), started in 2010 (Sorenson et al., 2019). Of these, only the CWC is Caribbean-specific, and it was initiated by BirdsCaribbean, in partnership with Wetlands International; checklists are stored in the eBird data repository (Sullivan et al., 2009). The data from the ISS, a geographically larger shorebird-specific monitoring scheme that includes Caribbean sites, are also available through eBird. Although not part of formal shorebird surveys, eBird has additional shorebird abundance data for the region as it is a repository for a large-scale citizen science project on bird abundances. Our objectives are to use the data from these formal survey efforts plus incidental data from eBird to identify important areas for shorebirds in the Caribbean, determine if any sites may qualify for special designation (i.e., WHSRN or IBA), and make recommendations about priorities for site protection and further research.

## Methods

Here we use ‘Caribbean’ to refer to the islands of the insular Caribbean; i.e., Greater and Lesser Antilles, The Bahamas, plus Trinidad and Tobago. The study area covers some 2.75 million km^2^, the archipelago consists of hundreds of subtropical and tropical islands, and includes 13 sovereign countries and 17 territories.

We broadly define shorebirds as species in the order Charadriiformes, excluding gulls (Sternidae) and auks (Alcidae). While our main interest is in shorebirds that are part of the Atlantic Flyway, we also included other North American migratory shorebirds in our study that are not traditionally considered reliant on the Atlantic Flyway (e.g., Black-necked Stilt (*Himantopus mexicanus*) if they were not categorized as rare in the Caribbean (Gerbracht & Levesque, 2019). We used as our taxonomic authority the Clements Checklist for taxonomic classification (Clements et al., 2019), which is also used by eBird. Exceptions include subspecies or geographic populations not listed by the Clements Checklist that are recognized by leading shorebird authorities (e.g., Andres et al., 2012); these are noted when reported. For a full list of species considered, and corresponding conservation status, see Table S2 (Supplemental Information). Special attention is given to focal species of the Atlantic Flyway Shorebird Initiative, which are those that are of high conservation concern, represent important habitat suites in the flyway, or have existing conservation plans (AFSI Business Plan, 2015). These species include American Golden-Plover (*Pluvialis dominica)*, American Oystercatcher (*Haematopus palliatus)*, Greater Yellowlegs (*Tringa melanoleuca)*, Lesser Yellowlegs (*T. flavipes)*, Piping Plover, Red Knot, Ruddy Turnstone (*Arenaria interpres)*, Sanderling (*Calidris alba)*, Semipalmated Sandpiper, Snowy Plover, Whimbrel (*N. phaeopus)*, and Wilson’s Plover. Three species are excluded from our analyses, Marbled Godwit (*Limosa fedoa*), Red-necked Phalarope (*Phalaropus lobatus*), and Purple Sandpiper (*C. maritima*); although they are AFSI focal species, they are listed as rare or very rare in the Caribbean region (Gerbracht & Levesque, 2019).

All data used in this assessment came from the World eBird Basic Dataset (Sullivan et al., 2009; publicly available at ebird.org/data/download). We downloaded the data from eBird on January 21, 2020 and filtered it using the R package ‘Auk’ (Strimas-Mackey, Miller & Hochachka, 2018) to include only records of interest (filtered by country and species; see 10.6084/m9.figshare.12760202 for data). The result was a list of all eBird shorebird records from January 1, 2010 through December 31, 2019 in the insular Caribbean. Duplicate checklists (i.e., those with the same Group Identifier), and records indicating presence but not abundance, were excluded. After these steps, the data were arranged by count, and counts over 750 for all species were reviewed for potential duplicates. For example, two observers could be birding together but fail to identify as a group when submitting their checklists.

With eBird data, as well as other survey protocols for unmarked individuals, it is impossible to sum only unique individuals at a site over a season, or even a single day, as individuals are not marked and turnover rates at each site are unknown. This means the same birds might be counted on the same or different days at the same site, and birds might move between sites. In addition, it is important to note that abundances in eBird are raw counts and not detection-corrected. Weather, habitat, season, effort, and species, among other factors, add variation to detectability (Johnston et al., 2014). Additional challenges that come with using eBird data are temporal bias (e.g., counting on weekends or during migration), spatial bias (e.g., counting in accessible areas or close to home), taxonomic bias (e.g., only counting preferred species), and issues with spatial precision (e.g., the nearest “hotspot” was selected instead of the exact location of the count). These challenges are briefly reviewed in the online document Best Practices for Using eBird Data (Strimas-Mackey et al., 2020; https://cornelllabofornithology.github.io/ebird-best-practices/; accessed 18 May 2020). Therefore, in our analyses, we mainly report species high-count data from a single site per season. Consequently, the values we report in our assessments are conservative in that they are minimum numbers of birds at each site at a single point in time. Our results are conservative also because, as mentioned, they do not represent cumulative birds over a season because insufficient data on site use and turnover rate prevent this assessment. Thus, what we report is a snapshot of the highest number of a species in each season/year/site.

CWC, ISS, and general eBird checklists used for this analysis differ in their methods. The CWC involves counting all birds found in wetlands following one of four protocols, with increasing levels of sophistication and intensity, carried out during the regional count period (three weeks in January/February) or anytime throughout the year (Sorenson et al., 2019), and many of the observers have received specialized training. A Level 1 protocol includes a basic count (a single count of birds present), while higher-level protocols include repeated counts (within 7 to 10 days), dual observers, or distance sampling; surveys can be point counts, transects, or area searches (Sorenson et al., 2019). The ISS employs volunteers to count shorebirds at known stopover sites every ten days for the duration of migration, encouraging counts during the same stage of the tide cycle at each site (https://www.manomet.org/wp-content/uploads/2018/03/ISS-Protocols_April2019.pdf). eBird data are referred to as semi-structured (Kelling et al., 2018); users are expected to report the survey protocol they used, the amount of effort per checklist (duration, distance, observers), and if the checklist is complete, i.e., the list reports all birds the surveyor was able to identify. In addition, the data pass through quality-assessment filters and checklists are flagged for expert review if they report aberrations, such as unusually high numbers of birds or a species outside of its normal range (Sullivan et al., 2009). CWC and ISS submissions are subject to these quality filters as well. Although eBird checklists are *ad hoc*, the data are valuable and may yield similar results to those from standardized surveys. When comparing eBird data to bi-monthly shorebird surveys with trained observers at Snooks Island, Florida, species richness and Shannon diversity index values were higher with eBird data, though there were not significantly different after accounting for survey effort (Callaghan & Gawlik, 2015). The authors suggested that eBird data at this location could substitute for standardized, more expensive surveys (Callaghan & Gawlik, 2015).

Our primary goal in this study was to identify important sites to shorebirds in the Caribbean. This can be accomplished through single-species counts, that is, the site is important to particular taxa, or through total counts at a site across all species, i.e., generally important to shorebirds. Subsequently, we used the results of these analyses to determine if sites would qualify for IBA or WHSRN status if they were not designated already.

### High counts for species

To determine important sites for each species, we identified the ten sites in the Caribbean with the highest counts of each species within each of three seasons. We defined seasons as Fall (August-November), Winter (December-February), and Spring (March-May), to coincide with primarily migratory and non-migratory time periods. We excluded summer months because migrants were largely absent. However, we did scan the summer months and included high counts in July as occurring in the Fall as such an occurrence would coincide with the biology of migration, rather than relying on the artificiality of the calendar; this resulted in one exception, a group of 2350 Lesser Yellowlegs observed on 31 July 2017. In cases where a single large site is comprised of a complex of wetlands that cannot be surveyed in a single visit, counts at unique sites across a three-day period were aggregated to derive the high count for that site (Sorenson & Gerbracht, 2014); the only site where this applied was Monte Cristi, Dominican Republic. However, there is always the chance that birds move between unique sites at wetland complexes; therefore, all counts from unique sites for multi-day data are provided in Data S1 and both the aggregated high count and the single highest count of all unique sites are reported when assessing population thresholds in tables. High-counts of a single individual were excluded. Sites did not repeat within the same season for the same year. That is, if the same wetland had the top 3 (for example) highest counts of a species, but all occurred in the Fall of 2010, we included the site only once on our list with the highest count of the three. If that same wetland had 3 of the highest counts but each in a different year, we included the site three times. In the event that there was a tie in abundance for the 10th place, we included all sites until the tie ceased. In addition, for each high count, we noted whether the checklist had an associated protocol type (i.e., CWC, ISS). Finally, we determined the current IBA status of sites where high counts were recorded by comparing the coordinates of those sites as entered on eBird with a shapefile of IBAs available from BirdLife International (http://www.datazone.birdlife.org/site/requestgis).

### Aggregated abundances for sites

Next, we determined which wetlands supported the highest abundances of shorebirds with all species aggregated. For this analysis, we included all Western Hemisphere shorebird species that are known to occur in the Caribbean (Gerbracht & Levesque, 2019) which included residents (e.g., Northern Jacana (*Jacana spinosa*)) and South American species (e.g., Collared Plover (*C. collaris*) in addition to North American migrants.

Currently, there is no way to easily distill bird abundances from eBird data over a geographic area such as a wetland complex. For example, organizing data by Locality ID, which is a unique identification number given to a pair of coordinates, may be useful; however, a checklist 5 m (for example, or any other distance) away at the same site could potentially have a different, non-consecutive Locality ID. The same can be true for organizing data by the Sampling Event Identifier, which is a unique number given to each checklist; if a site is sufficiently large, multiple checklists may have been completed by the same observer to cover the entire site.

Consequently, we investigated abundances of shorebirds by looking at the data in three different ways. First, we summed total shorebird counts for all species over the ten-year period by Locality ID. This provided a coarse starting point to identify sites with high shorebird abundances. For Locality IDs with more than 15,000 shorebirds over the ten-year period, we reviewed individual checklists and yearly abundances for the location to find peak values. If a Locality ID fell within an IBA, we reviewed all checklists that fell within the IBA boundaries. Second, we summed total shorebird counts by Sampling Event Identifier. This provided us with sites that had high numbers of shorebirds at one time. We report sites with more than 4,000 shorebirds in one visit as sites that require future, on the ground investigation for annual shorebird abundance surveys. Third, we visually examined the shorebird data mapped spatially in GIS with points representing total shorebird counts per checklist and displayed categorically by abundance bins. This allowed us to see clusters of high abundances spatially. We investigated data associated with the clusters by date.

### Special designation of identified sites

Both WHSRN sites and IBAs require minimum criteria to be met before designation (Donald et al., 2019; whsrn.org/why-whsrn/is-my-site-eligible/; accessed 18 May 2020). For example, if a site supports at least 1% of a geographic population of a species, it may qualify as an IBA (under criterion A4), and as a WHSRN site of Regional Importance; the latter designation is also given to those sites that support at least 20,000 shorebirds annually. If a site supports at least 10% of a geographic population and/or 100,000 shorebirds annually, it may qualify for designation as a WHSRN site of International Importance.

We determined the current IBA and WHSRN status of sites identified as supporting high numbers of a species or high aggregations of shorebirds by comparing the coordinates of those sites as entered on eBird with a shapefile of IBAs available from BirdLife International (http://www.datazone.birdlife.org/site/requestgis) and the two existing Caribbean WHSRN sites.

Finally, we compared observed high counts for species and aggregated abundances at each site with 1% and 10% of their estimated geographic population sizes to determine whether any sites exceeded these thresholds. That is, to determine if there are currently sites not yet designated by either organization that could qualify for inclusion. We also identified sites with abundances that made it to 0.95% levels, as sites to watch, with the thought that greater than 10 years of data might see years of higher abundance, or that detection-corrected or turnover-assessed analyses that might show the site meets the 1% cutoff. Unless otherwise noted, we used Andres et al. (2012) as a guide for global and regional shorebird population estimates. Since our counts are conservative—being slice of time, rather than across-year assessments—any indication a site qualifies for WHSRN or IBA should be viewed as being suitable at a minimum for that designation, and worth investigating further.

## Results

After data preparation, 211,013 shorebird observations in the insular Caribbean from 2010–2019 remained, representing 78,794 eBird checklists and cumulative total of 2.1 million shorebirds of 45 species. The area with the most checklists was Puerto Rico (22%; 17,414), followed by Cuba (13%; 9,935) and The Bahamas (∼12%; 9,402). St. Barthélemy had the fewest with just 29 checklists with shorebird records (0.03%). The months of September (28,770), October (22,344) and January (22,238) yielded the highest numbers of checklists over the decade, while the summer months of June and July yielded the lowest (both under 10,000). See Figs. S1 and S2 for summary graphs.

**Figure 1:**
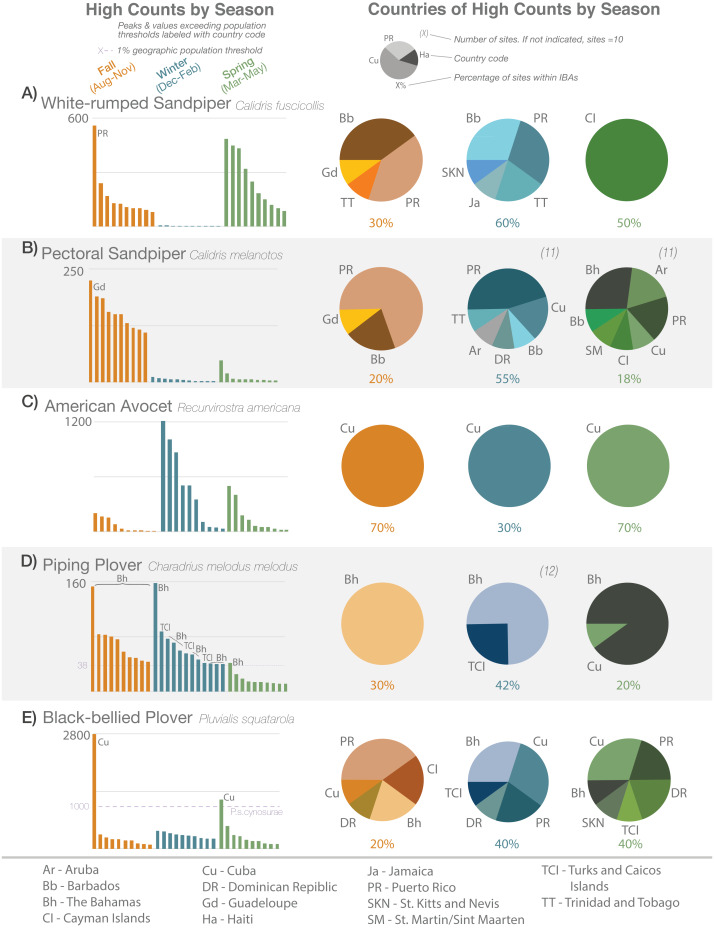
High-count records (with abundance and location, by season) of five species. Ranked single high-count records by abundance (*y*-axis) and season (bar graphs), and their proportional distributions by country and season (pie charts; same seasonal colors) for (A) White-rumped Sandpiper, (B) Pectoral Sandpiper, (C) American Avocet, (D) Piping Plover, and (E) Black-bellied Plover from eBird data 2010–2019 (see text for details). Dashed lines indicate 1% threshold of regional population size. Two-letter country codes indicate notable peak abundances or records exceeding thresholds in bar graphs, and distributions of records in pie charts; see legend at bottom of figure. There are 10 high-counts per season for each species except where indicated parenthetically above pie charts (occurred when multiple sites were tied in the 10th place of abundance). Percentages below pie charts indicate the proportion of sites that are within IBA boundaries each season. Sites may repeat in different years or seasons but not within the same season and year.

### High counts for species

High-count data revealed spatial and temporal patterns between and among species. For example, we observed seasonal differences in the highest counts of White-rumped Sandpiper (*Calidris fuscicollis*) vs Pectoral Sandpiper (*C. melanotus*; Figs. 1A and 1B). The White-rumped Sandpiper migrates through the Caribbean in the fall and spring while the Pectoral Sandpiper is primarily in the region in the fall. We also observed that while the high counts of White-rumped Sandpiper frequently occurred in Barbados and Puerto Rico in the fall and winter, the highest counts in the spring exclusively occurred in the Cayman Islands. The high counts for the Pectoral Sandpiper were mostly in Puerto Rico in the fall and winter, but also occurred in other countries. See Fig. S3 for seasonal/regional high-count graphs for all shorebird species in this study, and Data S1 for the eBird data that comprised these counts.

The high counts for some birds were widely distributed across the Caribbean, such as the Semipalmated Plover (*Charadrius semipalmatus*) for which the highest counts were recorded in five to seven countries, depending on the season. Other species’ highest counts occurred in a limited number of countries, such as the American Avocet (*Recurvirostra americana*), with highest counts only in Cuba, regardless of season (Fig. 1C) or Piping Plover (Fig. 1D), with highest counts only in The Bahamas, Cuba, and Turks and Caicos Islands.

Seasonal patterns were observed within the high-count data. As mentioned before, some species, such as Pectoral Sandpiper and American Golden-Plover pass through the Caribbean in the fall only, and do not over-winter. Other species, such as Killdeer (*C. vociferous*), are present year-round in the Caribbean but their numbers are augmented by migrants in the winter. White-rumped Sandpipers, on the other hand, are observed in their highest numbers in the fall and spring, but are virtually absent in the winter months (Fig. 1A).

We also observed for many species, instances of the highest count per season being several times higher than the next most abundant count. Examples of this were observed in one or more seasons for Black-necked Stilt, Black-bellied Plover (*P. squatarola*; Fig. 1E), Wilson’s Plover, Piping Plover (Fig. 1D), Western Sandpiper (*Calidris mauri*), Short-billed Dowitcher, Greater Yellowlegs and Red Knot; species not depicted in Fig. 1 are shown in Fig. S3.

Unsurprisingly, the high counts for species were not distributed evenly across the region. Generally, countries with larger areas (i.e., the Greater Antilles and The Bahamas) yielded more of the high-count records and had greater species richness. For example, of the 927 high-counts, 213 (∼23%) were in Cuba, representing 25 different shorebird species (Fig. S4). The greatest species richness (26) in the high-count data was found in Puerto Rico, with 189 of the high counts (∼20%). Interestingly, Guadeloupe and Barbados contributed almost equally to high-count records (46 and 42, respectively), yet the Barbados data included only eight species while the Guadeloupe data included fourteen. Similarly, high-count data from both Trinidad and Tobago and Turks and Caicos Islands represented 14 species, yet the former had three-fold more high-counts (79 vs. 26).

Of the 927 records that made up the high-count data, 715 (77%) were from general eBird checklists, 202 (22%) were from CWC protocols, and 10 (1%) were from ISS protocols (Fig. S5). General eBird checklists included the following protocol types: area, historical, incidental, random, traveling, and stationary. CWC protocol types included CWC Area Search, CWC Point Count, and CWC Traveling Count. There was only one International Shorebird Survey protocol type.

A few species included in our study were never recorded in high numbers (>30 individuals) across the entire decade. These included American Oystercatcher (highest count 17), Hudsonian Godwit (*L. haemastica*; 30), Upland Sandpiper (*Bartramia longicauda*; 21), and Wilson’s Phalarope (*P. tricolor*; 5).

Nearly 54% (501 records) of the high counts occurred at sites that are within IBA boundaries. The percentage of high counts occurring within an IBA varied by season and by species. In the case of the American Avocet and American Golden-Plover (Figs. 1D and 1E), when bird abundances were largest (in the winter for the avocet and the fall for the golden-plover), high counts were less likely to be recorded within an IBA. For other species, such as the Stilt Sandpiper (*Calidris himantopus*) and Whimbrel, high counts occurred in IBAs more than 60% of the time, regardless of season.

Regarding IBA assignment within eBird data, when eBird data are downloaded, a column is generated called “IBA Code.” Ostensibly, when a checklist falls within the boundary of an IBA, it is assigned the corresponding BirdLife International code. The eBird Basic Dataset Metadata (v1.12; bundled with files upon download) does caution that “IBA information is currently only available for some countries.” For the 927 records that we geospatially compared with IBA boundaries, however, we came across several instances of records occurring in an IBA but not receiving an IBA code. There were two reasons for this. First, two IBAs in The Bahamas were not recognized by eBird: Kemp Cay to Pigeon Cay and Joulter Cays, both designated in 2012. Second, for other cases it appears there was spatial error when assigning coordinates to a checklist. Sometimes, the checklist location would be a matter of meters outside the IBA boundary. We reviewed each set of coordinates and determined whether the checklist qualified as occurring within an IBA based on the location of the checklist, the IBA boundary, species observed, and surrounding habitat (see Figs. S6A and S6B for examples).

### Aggregated abundances for sites

**Figure 2:**
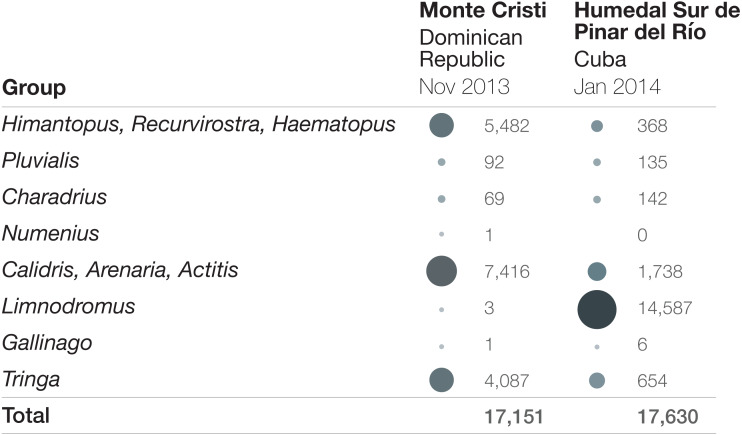
Bird abundance comparison by taxonomic group at Monte Cristi, Dominican Republic and Humedal Sur de Pinar del Río, Cuba. Surveys were completed in November 2013 (Dominican Republic) and January 2014 (Cuba). Increasing circle sizes and darkness represent relatively larger proportions of the total abundance.

Some sites showed up consistently across years as important sites for shorebirds, while other sites had only one or two years of very high numbers. There were two particularly notable sites. The first is the wetlands in the IBA Humedal Sur de Pinar del Río, Cuba (also referred to as “Humedal Sur de Los Palacios”), which consistently reported the highest aggregations of shorebirds in the Caribbean. In 2012, a total of 6,964 shorebirds were counted in just 3 checklists, each from a different season. In 2013, three checklists, again, all in different seasons, yielded 22,354 shorebirds. Just one checklist in January 2014 reported 17,630 individuals. These high abundances were largely driven by aggregations of Short-billed Dowitchers, in some cases exceeding 10,000 birds (Fig. 2). Though on other occasions, large aggregations of shorebirds were reported for other species at the site, including 4,287 Least Sandpipers (*C. minutilla*), 1,205 Black-bellied Plovers, and 740 Black-necked Stilts.

The second site is the Monte Cristi wetland complex in the Dominican Republic, which also consistently supports high abundances of shorebirds. Over the course of a three-day survey in November 2013 of the different wetlands that make up this large complex, 17,151 shorebirds were recorded. The large cumulative count at this site was driven by several species, including Black-necked Stilt (highest count = 5,480), Lesser Yellowlegs (3,219), Semipalmated Sandpiper (3,139), and Stilt Sandpiper (2,213). It is notable that even though overall numbers are similar to the highest count in Cuba, and the surveys were only months apart, it is clear that the survey data do not merely represent a shift of birds between sites over time, because the abundances of the taxa are so different (Fig. 2; numbers by species can be found in Table S3). Counts of over 4,000 shorebirds were recorded at Monte Cristi on three other 3-day surveys: January 2019: 6,473 shorebirds; October 2012: 5,325 shorebirds; February 2015: 4,374.

**Table 1:** Observations that surpass geographic population thresholds. Single records that exceed 1% of the geographic population threshold for a species. Only the highest count for each site per season per year is included. Sites designated as IBAs are indicated with IBA code; none are WHSRN sites. See Data S1 for more details of each record.

**Species**	**Estimated Population**^a^	**1% threshold**	**High count**	**% of Pop.**	**Country**	**Site** *(Island)*	**IBA** **code**	**Date**
Black-bellied Plover *Pluvialis sqatarola cynosurae*^b^	100,000	1,000	2,800	2.8	Cuba	Laguna de la Jaiba	CU008	10-Oct-2019
Black-bellied Plover	100,000	1,000	1,205	1.2	Cuba	Humedal Sur de Pinar del Río	CU003	22-Mar-2012
Wilson’s Plover *Charadrius wilsonia wilsonia*	14,000^c^	140	150	1.1	Cuba	Humedal Sur de Pinar del Río	CU003	22-Jan-2013
Wilson’s Plover *C. w. cinnamominus*	7,500^c^	75	275	3.7	Bonaire	Harbour Village		20-Sep-2013
Piping Plover *C. melodus melodus*	3,760^d^	38	158	4.2	The Bahamas	Ambergris Cay Group *(Berry Islands)*	BS042	5-Feb-2017
			153	4	The Bahamas	Pigeon Cay *(Berry Islands)*	BS042	31-Oct-2016
			84	2.2	The Bahamas	Kemp’s Cay Flats *(Berry Islands)*		31-Oct-2016
Piping Plover	3,760	38	88	2.3	Turks and Caicos Islands	Cay off East Caicos	TC005	17-Jan-2017
			42	1.1	Turks and Caicos Islands	Cay off East Caicos	TC005	30-Jan-2016
			56	1.5	Turks and Caicos Islands	Cay off East Caicos	TC005	20-Jan-2019
Piping Plover	3,760	38	83	2.2	The Bahamas	Joulter Cays *(North Andros)*	BS041	29-Sep-2015
			80	2.1	The Bahamas	Joulter Cays *(North Andros)*	BS041	12-Nov-2017
			54	1.4	The Bahamas	Joulter Cays *(North Andros)*	BS041	1-Feb-2017
Piping Plover	3,760	38	77	2	The Bahamas	Moriah Harbour Cay *(Exuma)*		1-Feb-2016
Piping Plover	3,760	38	76	2	The Bahamas	Young Sound *(North Andros)*		18-Nov-2016
			43	1.1	The Bahamas	Young Sound *(North Andros)*		19-Oct-2013
Piping Plover	3,760	38	71	1.9	The Bahamas	Kamalamae Cay *(North Andros)*		4-Feb-2016
			58	1.5	The Bahamas	Kamalamae Cay *(North Andros)*		16-Nov-2016
			42	1.1	The Bahamas	Kamalamae Cay *(North Andros)*		9-Mar-2013
			40	1.1	The Bahamas	Kamalamae Cay *(North Andros)*		7-Feb-2017
			40	1.1	The Bahamas	Kamalamae Cay *(North Andros)*		23-Feb-2011
Piping Plover	3,760	38	60	1.6	The Bahamas	Freeport Aggregate *(Grand Bahama)*		6-Jan-2019
Piping Plover	3,760	38	50	1.3	The Bahamas	Island Homes Beach *(Abaco)*		14-Nov-2015
			49	1.3	The Bahamas	Island Homes Beach *(Abaco)*		12-Nov-2016
			45	1.2	The Bahamas	Island Homes Beach *(Abaco)*		18-Nov-2017
			41	1.1	The Bahamas	Island Homes Beach *(Abaco)*		18-Jan-2016
Piping Plover	3,760	38	47	1.2	The Bahamas	Cherokee Sound *(Abaco)*		1-Jan-2019
Red Knot *Calidris canutus rufa*	42,000	420	410	0.98	Turks and Caicos Islands	Caicos Banks/ Blue Hole		20-Jan-2017
Short-billed Dowitcher *Limnodromus griseus griseus/hendersoni*	78,000	780	14,517	18.61	Cuba	Humedal Sur de Pinar del Río	CU003	30-Jan-2014
			12,000	15.4	Cuba	Humedal Sur de Pinar del Río	CU003	26-Sep-2019
			8,397	11.5	Cuba	Humedal Sur de Pinar del Río	CU003	24-Nov-2013
			2,833	3.6	Cuba	Humedal Sur de Pinar del Río	CU003	15-Mar-2013
			1,985	2.5	Cuba	Humedal Sur de Pinar del Río	CU003	22-Jan-2013
			800	1	Cuba	Humedal Sur de Pinar del Río	CU003	10-Dec-2019
Short-billed Dowitcher	78,000	780	5,000	6.4	Cuba	Cayo Guilleremo		25-Feb-2018
			1,000	1.3	Cuba	Cayo Guilleremo		4-Mar-2018
			850	1.1	Cuba	Cayo Guilleremo		16-Feb-2011
			800	1	Cuba	Cayo Guilleremo		6-Jan-2017
Short-billed Dowitcher	78,000	780	1,500	1.9	Turks and Caicos Islands	Caicos Banks/ Blue Hole/Black Rock		20-Jan-2017
			770	0.99	Turks and Caicos Islands	Caicos Banks/ Blue Hole/Black Rock		30-Jan-2019
Short-billed Dowitcher	78,000	780	955	1.2	The Bahamas	Joulter Cays *(North Andros)*	BS041	29-Sep-2015
Short-billed Dowitcher	78,000	780	759	0.97	Cuba	Las Salinas, Zapata Swamp	CU006	19-Jan-2012
Short-billed Dowitcher	78,000	780	750	0.96	Cuba	Punta Hicacos, Veradero		25-Jan-2012

**Notes.**

a From Andres et al. (2012) unless otherwise noted.

b Subspecies or geographic population not listed in The Clements Checklist Clements et al. (2019).

c Based on Zdravkovic (2013).

d Based on US Fish and Wildlife Service (2019).

There were two additional sites that require future investigation of annual shorebird abundances as more than 4,000 shorebirds were recorded in one visit at each. In February 2018, a checklist from Cayo Guillermo on the north coast of Cuba was reported with 5,107 shorebirds, with a flock of 5,000 Short-billed Dowitchers making up the bulk of the count. In February 2010, Old Harbour Bay in Jamaica hosted 4,818 shorebirds, including 4,000 Least Sandpipers and 600 Lesser Yellowlegs.

**Figure 3:**
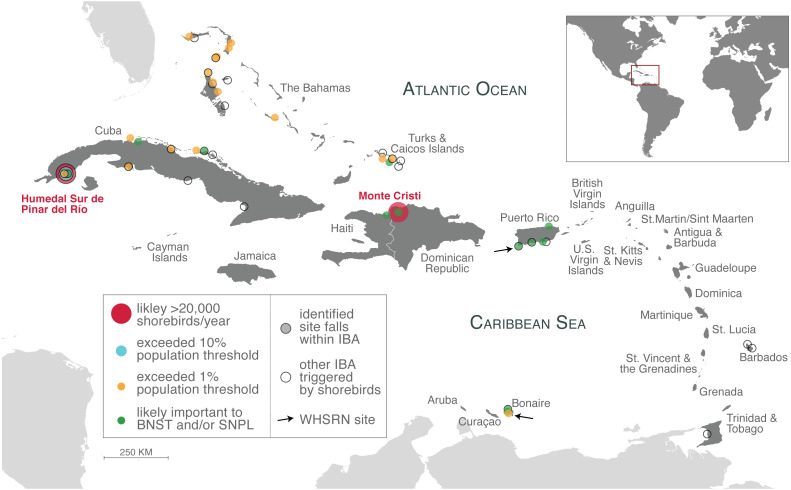
Important shorebird sites in the insular Caribbean. Sites identified from our analysis as likely supported more than 20,000 shorebirds annually (large red circles), exceeded 10% population thresholds (light blue circle), exceeded 1% population thresholds (orange circles; counts that meet or exceed 0.95% –two sites in Cuba –also included). Sites that are likely important to Black-necked Stilts (BNST) and/or Snowy Plover (SNPL), but threshold values could not be determined (green circles), are also indicated. All sites are listed in Tables 1–3. Identified sites that fall within an IBA are outlined in black. Approximate location of other IBAs not identified in our study, but were triggered by shorebird species (Table S1), are indicated with an unfilled circle and black outline. Arrows indicate the location of two WHSRN sites. The islands of the insular Caribbean are in dark gray; continental areas are in light gray.

### Special designation of identified sites

We identified several sites that are potential WHSRN sites or IBAs based on either cumulative shorebird abundance or on exceeding population thresholds for particular species, even counting birds on only a single survey (Table 1; Fig. 3). Based on cumulative shorebird abundance, Humedal Sur de Pinar del Río in Cuba supports more than 20,000 shorebirds annually and it is very likely that the Monte Cristi site in the Dominican Republic does as well. Consequently, both sites may meet the WHSRN abundance criterion for a site of Regional Importance. Humedal Sur de Pinar del Río is already designated as an IBA but not as a WHSRN site; Monte Cristi does not have any international designations.

**Figure 4:**
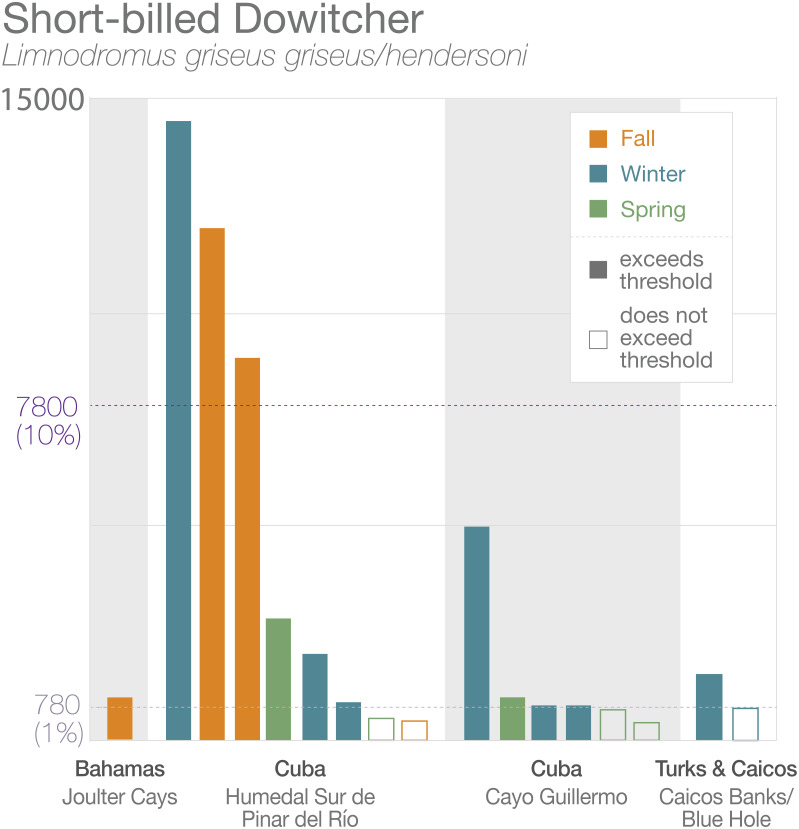
High-count data at four sites that exceeded threshold limits for Short-billed Dowitchers (*Limnodromus griseus griseus/hendersoni*) by season. The top dashed line indicates 10% of the population for the combined subspecies and the bottom dashed line indicates 1%. Bars are color-coded by season. Solid bars represent records that exceeded threshold limits, unfilled bars are records that did not.

Importantly, some sites may support more than 1% of the geographic population of more than one species. In the case of Humedal Sur de Pinar del Río, the site has been recorded on three occasions to support more than 10% of the geographic population of Short-billed Dowitchers (*L. g. griseus/hendersoni;* >7,800 birds), which would meet the criterion for a site of International Importance, a designation that currently does not exist in the Caribbean region (Fig. 4). In addition, on single surveys the site has been recorded to support 1.2% (1,205 birds) of Black-bellied Plovers, if these birds are the debated *cynosurae* subspecies, and 1.1% (150 birds) of Wilson’s Plovers (*C. w. wilsonia*).

We also found that Joulter Cays, part of an IBA in The Bahamas, has single-survey records of more than 1% of the Short-billed Dowitcher population (955 birds or 1.2%) and on two occasions more than 1% of the Piping Plover population (83 birds or 2.2%; 80 birds or 2.1%). A sandbar and rock formation south of Middle Caicos in Turks and Caicos Islands hosted 1.9% (1,500 birds) of the Short-billed Dowitcher population and another year 0.99% (770 birds), in addition to 410 Red Knots (0.98% of the *rufa* population). Two other sites may be important to Short-billed Dowitchers, as they were close to the 1% threshold from just a single count: Las Salinas in Zapata Swamp, Cuba (759 birds or 0.97%) and Punta Hicacos in Veradero Cuba (750 birds or 0.96%). These and subsequent records are summarized in Table 1.

The cays off the northern coast of Cuba are another area where large numbers of shorebirds have been documented. Cayo Coco, part of an IBA, has been recorded on a single survey to support 3,500 Black-necked Stilts. Cayo Guillermo, the next cay to the west, and separated from Cayo Coco by a narrow inlet, does not fall within the IBA boundaries but has on three occasions supported more than 1% of the population of Short-billed Dowitchers. Farther to the east on the northern coast of mainland Cuba, 2.8% of the population of Black-bellied Plovers (2,800 birds) were recorded at one time at Laguna de la Jaiba in Villa Clara Province.

Both The Bahamas and Turks and Caicos Islands reported high numbers for Atlantic Piping Plover. In addition to Joulter Cay, Piping Plover crossed the 1% threshold three times on the Berry Islands. Twice in an IBA (158 birds or 4.2%; 153 birds or 4%) and once on Kemp’s Cay just outside of the IBA boundary (84 birds or 2.2%). Just over two percent of the population (88 birds) was observed in the East Caicos IBA in Turks and Caicos Islands and over 1% was seen at the same location on two other occasions. Other sites that exceeded the population threshold for Atlantic Piping Plover were located on the Bahamian islands of Exuma, Andros, Grand Bahama, and Abaco (see Table 1).

Finally, a large group of Wilson’s Plovers (275 birds) was observed at Harbour Village in Bonaire. If these birds are from the *C. w. cinnamominus* subspecies, this represents 3.7% of the population. The 1% threshold for the nominate subspecies *C. w. wilsonia* is 140 birds, so even if this group was a mixed flock of both subspecies, which is doubtful based on geography (Zdravkovic, 2013), it would still meet the threshold limits.

We did not include Snowy Plover in Table 1 as its taxonomy is debated so it required a more nuanced treatment (see Discussion); but there are sites in the Caribbean that appear to be important for this species. In 2012, it was estimated that 200 Caribbean *C. n. tenuirostris* existed, compared to 25,800 continental *C. n. nivosus* birds (Andres et al.), though that number is more likely around 950 individuals (A Lesterhuis, pers. comm., 2020). Therefore, 1% of the *tenuirostris* race could be just 10 individuals. In our analysis, we identified 45 records with 10 or more Snowy Plovers from 8 countries (see Data S1); this includes 17 records from the summer months of June and July. However, it remains unclear what proportion of the counts are *tenuirostris* vs. *nivosus*. We present the highest counts (20 or more birds) for Snowy Plover at the species level (*Charadrius nivosus*) in Table 2. In addition to Bonaire, Cuba, the Dominican Republic, Haiti, Puerto Rico, and Turks and Caicos Islands, two other countries, The Bahamas (particularly the island of Inagua) and Anguilla (Cove Pond and Long Salt Pond) had consistent sightings of Snowy Plover, though never more than 20 at one time. In addition to the sites listed in Table 2, these areas should be considered for further investigation for Snowy Plover populations in the Caribbean.

**Table 2:** Sites with high abundances of Snowy Plovers (*Charadrius nivosus*). Records of 20 or more Snowy Plovers, by country. Note, only the highest single count for each site per season per year is included. High counts at Monte Cristi represent cumulative counts over 2-day survey period with the highest single checklist count in parenthesis. Sites designated as IBAs are indicated with IBA code. There are an estimated 950 birds of the Caribbean race *C. n. tenuirostris* (A Lestershuis pers. comm., 2020). See Data S1 for all records of 10 or more Snowy Plovers.

**High Count**	**Country**	**Site**	**IBA** **code**	**WHSRN site**	**Date**
41	Bonaire	Salina Matijs	AN009		30-Oct-2019
20	Bonaire	Salina Matijs	AN009		12-Oct-2018
29	Bonaire	Bopec/Gotomeer			4-Sep-2015
20	Bonaire	Bopec/Gotomeer			14-Sep-2019
40	Haiti	Fort Liberte Bay			19-Nov-2015
33	Cuba	Humedal Sur de Pinar del Rio	CU003		30-Jan-2014
21	Cuba	Salinas de Bido			16-Dec-2015
33	Puerto Rico	Cabo Rojo	PR008	yes^a^	30-Dec-2012
30	Puerto Rico	Piñones			10-Nov-2017
27	Puerto Rico	Cabo Rojo	PR008	yes^a^	31-Jan-2011
20	Puerto Rico	Cabo Rojo	PR008	yes^a^	5-Aug-2018
20	Puerto Rico	Cabo Rojo	PR008	yes^a^	27-Dec-2014
20	Puerto Rico	Reserva Natural Las Cucharas	PR011		23-Mar-2019
22 *(19)*	Dominican Republic	Monte Cristi			12-Sep-2016
21	Turks and Caicos Islands	South Caicos Salinas Pond East			3-Nov-2013

**Notes.**

a Site of Regional Importance.

**Table 3:** Sites with high abundances of Black-necked Stilts (*Himantopus mexicanus*). Records of 1,000 or more Black-necked Stilts, by country. Note, only the highest single count for each site per season per year is included. High counts at Monte Cristi represent cumulative counts over 3-day survey period with the highest single checklist count in parenthesis. Sites designated as IBAs are indicated with IBA code. There are an estimated 175,000 North American Black-necked Stilts (Andres et al., 2012) and 38,500 Caribbean resident birds (A Lesterhuis, pers. comm., 2020). See Data S1 for all records of 385 or more Black-necked Stilts.

**High Count**	**Country**	**Site**	**IBA** **code**	**WHSRN site**	**Date**
5,480 *(2,700)*	Dominican Republic	Monte Cristi			20-Nov-2013
1,986 *(1,700)*	Dominican Republic	Monte Cristi			25-Feb-2015
1,732 *(1,500)*	Dominican Republic	Monte Cristi			3-Oct-2015
3,500	Cuba	Cayo Coco	CU012		30-Jan-2018
1,000	Cuba	Cayo Coco	CU012		17-Jan-2019
2,425	Puerto Rico	Cabo Rojo	PR008	yes^a^	17-Dec-2016
2,000	Puerto Rico	Cabo Rojo	PR008	yes^a^	25-Aug-2012
2,000	Puerto Rico	Humedal Juaca/ Bosque de Aguirre			11-Dec-2011
1,500	Puerto Rico	Cabo Rojo	PR008	yes^a^	30-Dec-2013
1,000	Puerto Rico	Cabo Rojo	PR008	yes^a^	5-Aug-2018
1,000	Puerto Rico	Cabo Rojo	PR008	yes^a^	24-Nov-2012

**Notes.**

a Site of Regional Importance.

We also did not include Black-necked Stilt or Killdeer in Table 1 for similar reasons. The Monte Cristi wetlands in the Dominican Republic are an important site for Black-necked Stilts, with large numbers repeatedly observed (5,480; 1,986; 1,732; Table 3). The salt ponds in Cabo Rojo, Puerto Rico also hosted large numbers (2,425; 2,000) of Black-necked Stilts on two occasions, though this is already an IBA and WHSRN site. A second site in Puerto Rico that is important to this species is Humedal Juaca, which was recoded on one occasion to have 2,000 birds. One percent of the North American Black-necked Stilt population is 1,750 individuals (Andres et al., 2012) though the Caribbean population is resident, so this threshold is not appropriate (Robinson et al., 2020). Approximately 38,500 resident Black-necked Stilts are estimated to live in the Caribbean (A Lesterhuis, 2020, pers. comm.). Thus, the large numbers of Black-necked Stilts observed at these sites could represent more than 1% of either, or both, populations. We identified 66 records with 385 (1% of the Caribbean population) or more Black-necked Stilts from Cuba, Dominican Republic, Haiti, Jamaica, and Puerto Rico (see Data S1); this includes 2 summer records.

We found six records of more than 100 Killdeer, all occurring during the winter months: the West End of Grand Bahama, The Bahamas, in three different years with numbers as high as 232; Embalse Niña Bonita, Cuba; and South Beach of New Providence, The Bahamas in two different years. It remains unclear if the numbers we observed in these winter high-counts were due to seasonal concentrations of local birds or augmentation from the mainland (Jackson & Jackson, 2020).

## Discussion

### The insular Caribbean is an overlooked but important region for migratory shorebirds

The Atlantic Flyway Shorebird Initiative classifies the Caribbean as one of its seven priority geographies and recognizes the urgent need to expand shorebird conservation participation in the region (AFSI Business Plan, 2015). Despite its clear importance to a number of shorebird species, however, the Caribbean is often overlooked or underappreciated when addressing North American shorebird conservation. To our knowledge, this study is the first Caribbean-wide assessment of shorebird use. The only prior assessment of the region that we know of is a white paper from 2014 that includes the first five years of the CWC data, so it is limited to those protocols and includes under 4,000 checklists (Sorenson & Gerbracht, 2014).

There are many factors that might contribute to the Caribbean’s lack of attention within the Atlantic Flyway, such as lower overall shorebird abundance, the geopolitical nature of the region, and knowledge gaps. First, the Caribbean does not support the spectacular numbers of migrating shorebirds seen in some other areas of world, like the nearly one million shorebirds in the Bay of Panama (Buehler, Castillo & Angehr, 2002). Second, the unique geography of hundreds of islands combined with dozens of different political institutions poses a challenge to coordinated monitoring and reporting efforts. Third, few studies investigate shorebird use of Caribbean islands, and those that do tend to focus mainly on southern Puerto Rico (e.g., Parks, Collazo & Ramos-Alvarez, 2016a; Parks, Collazo & Ramos-Alvarez, 2016b; Wunderle, Waide & Fernandez, 2009) and Cuba (e.g., Nol et al., 2014), or specifically on Piping Plover (e.g., Johnson et al., 2018; Gratto-Trevor et al., 2016).

Encouragingly, some knowledge gaps about Caribbean shorebirds are starting to close. In a 1985 paper about the status and distribution of Piping Plovers, it was noted that support for its distribution in the Caribbean was “sketchy at best” (p. 343) and cited a personal communication for suitable habitat in Cuba (Haig & Oring, 1985). However, with the continued expansion of the International Piping Plover Census in the Caribbean, The Bahamas is now recognized as a major wintering area for the species, representing 27% of all birds counted in the census (Gratto-Trevor et al., 2016).

In our study, we identified two major priority areas for shorebirds both by overall abundance and for individual species. First, the Humedal Sur de Pinar del Río in Cuba very likely exceeds 20,000 shorebirds annually and breaks IBA and WHSRN species-abundance thresholds for the Short-billed Dowitcher (>10%), Black-bellied Plover (>1%; assuming *P. s. cynosurae*), and Wilson’s Plover (>1%). Indeed, this site is known for its high species richness and waterbird abundance and has been identified as one of the most important sites for waterbird conservation on Cuba’s southern coast (Aguilar et al., 2019). It is also a site of illegal waterbird hunting, which Aguilar et al. (2019) cite as a main threat to the site. While the area is designated as an IBA, it is not a WHSRN site. Nationally, the Cuban Centro Nacional de Áreas Protegidas (CNAP) proposed the site for legal protection in 2013 as a wildlife refuge (to be known as Refugio de Fauna Humedal Sur de Los Palacios) of local, although not national, significance (Centro Nacional de Areas Protegidas, 2013), though the proposal has yet to be approved. The second site, the Monte Cristi wetland complex in the Dominican Republic also likely exceeds 20,000 shorebirds annually and regularly hosts large numbers of Black-necked Stilts. It is listed as a National Park but has no other designations of which we are aware. The Dominican Republic does have 21 other sites that are designated IBAs, yet nearly one-third of those are classified as “in danger”, and they are the only IBAs out of the 294 in the Caribbean to receive such a classification, due to high pressure threats such as habitat loss and hunting/trapping (datazone.birdlife.org/country/dominican-republic/ibas; accessed 18 May 2020).

Two other sites that we think require further on-the-ground investigation because they both have hosted more than 4,000 shorebirds in a single day are Cayo Guillermo in Cuba, and Old Harbour Bay in Jamaica. Jamaica represented 50% of high-count winter data for Least Sandpipers, with the highest single-survey count at 4,000 birds (the 1% threshold is 7,000 individuals). With increased monitoring and/or studies that determine turnover rate, the importance of these sites may be revealed.

We identified 15 additional sites that might meet population threshold criteria for designation as an IBA, WHSRN site, or both (Table 1). This number excludes sites that support high abundances of Snowy Plovers or Black-necked Stilts (but see Tables 2 and 3) and Killdeer for the reasons presented in the following section. While designation of an IBA does not reveal information about protected status of the site, the IBA criteria, according to BirdLife International, provide a common currency by which sites can be evaluated and compared, and to ensure that sites are significant for international conservation of birds (http://www.datazone.birdlife.org/site/ibacriteria; accessed 18 May 2020). However, identification of IBAs can catalyze conservation and management actions at sites and have an impact on national conservation policy (Waliczky et al., 2019). WHSRN sites, however, are not designated solely on quantitative factors. Landowners must agree to make shorebird conservation a priority, protect and manage the site for shorebirds, and report annually to the WHSRN Executive Office (whsrn.org/why-whsrn/is-my-site-eligible/; accessed 18 May 2020).

The IBA criterion A4 for congregations states that the population thresholds must be met “on a regular or predictable basis” though no definitions of regular or predictable are provided (Donald et al., 2019). We would like to emphasize that sites in the Caribbean may be flexible in importance to shorebirds in that they could be important in some years but not others. Shifting resources such as changes in water level and food availability are known to affect shorebird abundance and distribution (Collazo et al., 1995; Mathot, Smith & Elner, 2007). Our data represent just a 10-year assessment of the region, and while there are some sites that clearly are used repeatedly by shorebirds during this period, others may lack sufficient data and require further investigation to determine degree of consistent use.

There are 21 IBAs in the Caribbean that were triggered by the presence of shorebirds (Table S1). Fourteen of those sites did not reveal themselves in our analysis, and of the seven sites that overlapped, only two were identified because of the same species. For example, both Joulter Cays and Kemp Cay to Pigeon Cay were identified by BirdLife and our assessments as important to Piping Plovers; they were the only species in our analysis to cross population thresholds at these sites. For Humedal Sur de Pinar del Río, BirdLife and our study identified it as important because of Short-billed Dowitchers (though the count in our analysis is 4,500 birds greater than the BirdLife estimate), but our analysis also suggests that the site is important to Wilson’s Plovers and Black-bellied Plovers. Our analysis also revealed three IBAs that were not listed as having a shorebird trigger species, though they seem to qualify based on numbers: Zapata Swamp in Cuba (Table 1), as being potentially important to Short-billed Dowitchers, and Reserva Natural Las Cucharas and Salina Matijs in Bonaire (Table 2) for hosting high numbers of Snowy Plover. We provide an aggregated list of the IBAs triggered by shorebird species, WHSRN sites, and novel sites identified in this study (from Tables 1–3) in Data S2.

One popular designation that we have not mentioned thus far are wetlands of international importance under the Ramsar Convention (Ramsar, 2013). Some IBA criteria were designed to align with particular Ramsar criteria; for example, IBA Criterion A4 on congregations align with Ramsar criteria 5 (a wetland that regularly supports 20,000 or more waterbirds) and 6 (a wetland that regularly supports 1% of the individuals in a population of one species or subspecies of waterbird) (Ramsar, 2013; Waliczky et al., 2019). Neither the Humedal Sur de Pinar del Río site in Cuba nor the Monte Cristi site in the Dominican Republic are designated as wetlands of international importance under the Ramsar Convention. However, the website of Cuba’s CNAP states a proposal is underway to nominate the site (http://www.snap.cu/index.php/ct-menu-item-10/ct-menu-item-12; accessed 18 May 2020). Similar to WHSRN stakeholder buy-in, Ramsar site designation is not just a paper formality. Designated Ramsar sites are supposed to be managed to maintain ecological character, and retain essential functions and values (Ramsar, 2013). In 2009, Ramsar formed the Regional Initiative for the Conservation and Wise Use of Caribbean Wetlands, also called CaRIWet or Caribbean Wetlands Regional Initiative, to facilitate the implementation of the Ramsar Convention in the Caribbean (ramsar.org/activity/ramsar-regional-initiatives; Accessed 20 June 2020). We note that someone seeking information should search under all three initiative names because some reports are linked only to a single name.

### Knowledge gaps in subspecies data hinder conservation recommendations

Snowy Plover taxonomy is currently under debate. While some authorities only recognize two subspecies (*C. n. nivosus* and *C. n. occidentalis*; e.g., Clements et al., 2019; Gill, Donsker & Rasmussen, 2020), others recognize a third, *C. n. tenuirostris* (Andres et al., 2012; US Fish and Wildlife Service, 2011; Funk, Mullins & Haig, 2007). The *tenuirortris* subspecies was originally thought to include mainland Gulf of Mexico and Caribbean birds, though recent genetic work suggests that continental birds are more similar to *nivosus* and that the *tenuirostris* group is limited to the Caribbean (D’Urban, Jackson & Jackson, 2020). Unfortunately, there are no strong morphological differences between these two subspecies, leading them to be indistinguishable in the field, and little information exists about range overlap during migration (Page et al., 2020).

Snowy Plover are year-round breeding residents on some islands in the Caribbean and are known to occur in the southern Bahamas, Cuba, Dominican Republic, Haiti, Puerto Rico, Turks and Caicos Islands, Cayman Islands, Anguilla, the US and British Virgin Islands, St. Barthélemy, St. Kitts and Nevis, Antigua and Barbuda, Guadeloupe, Grenada, Aruba, Bonaire, Curaçao, and Tobago (Gerbracht & Levesque, 2019; Elliott-Smith et al., 2004). Though consistent nesting was recorded for Snowy Plover on St. Martin since 1959, no birds or nests were observed during annual surveys from 2005–2010, leading some to believe the species extirpated (Brown, 2012); however, 1–2 individuals were recorded there in eBird in 2016, 2017, and 2019 (Nguyen, 2019; Van es, 2017; Fischer, 2016).

Recent genetic studies suggest that the *C. n. tenuirostris* population of Snowy Plovers differs sufficiently from *C. n. nivosus* and they should be considered separate conservation units (Funk, Mullins & Haig, 2007; D’Urban, Jackson & Jackson, 2020). The majority of tissue samples in these studies, however, originated from Puerto Rico; there were two samples each from Turks and Caicos Islands, Bermuda, and Haiti, and none originated from other places Snowy Plovers occupy. The migratory movements of Snowy Plover are poorly known within the Caribbean, in addition to limited understanding of movements from the mainland to the Caribbean (Gorman & Haig, 2002; Page et al., 2020). It is critical to identify where *C. n. tenuirostris* persists, their complete range, and where they overlap with *nivosus* to conserve the subspecies. Finally, D’Urban Jackson et al. (2020) identified that the eastern United States *nivosus* population also warrants its own conservation unit status. This is important because migratory mainland birds that use the Caribbean would originate from this unique population.

Black-necked Stilts are common breeding residents in the Caribbean across the southern Bahamas, Greater Antilles, and Virgin and Cayman Islands; they are uncommon in the northern Bahamas and are rarely seen south of Guadeloupe (Raffaele et al., 2020). They are purported to be short to medium-distance continental migrants from the USA to Mexico and Central America (Robinson et al., 2020). To our knowledge, there have been no tracking studies completed that describe regular stilt movements between the North American mainland and the Caribbean, though such movements are certainly possible. In a banding study of the Endangered Hawaiian subspecies *H. m. knudseni*, interisland movements up to 295 km were reported (Reed et al., 1998). It is unknown if or how many North American Black-necked Stilts use the Caribbean, which islands they use, or their arrival and departure dates. Nevertheless, the sites identified from our analysis (Table 3) with the highest records of Black-necked Stilts—Monte Cristi, Dominican Republic; Cayo Coco, Cuba; and Cabo Rojo, Puerto Rico—are likely to represent more than 1% of either, or both, of the North American and Caribbean populations.

The Caribbean Killdeer subspecies, *C. v. ternominatus,* is a resident to The Bahamas, Greater Antilles and the Virgin Islands (Jackson & Jackson, 2020). However, there are no reliable population size estimates for this subspecies. Wetlands International (2020) estimates between 1–10,000 birds as a “best guess” and suggests a 1% population threshold of 100, which assumes the upper limit of the range. The estimated population for the North American subspecies, *C. v. vociferous,* is orders of magnitude larger than that of the Caribbean, at one million birds (Andres et al., 2012). Without a reliable population estimate for the *ternominatus* subspecies or the ability to extract its abundance from potentially mixed groups during migratory months, we unfortunately could not justify its inclusion in our results.

There is only a single population estimate for the combined eastern subspecies of Short-billed Dowitcher (*griseus* and *hendersoni*), however, only the nominate subspecies is reported as migrating through the West Indies while *hendersoni* tends towards Central America (Jehl Jr et al. , 2020) (see Cartwright (2019) for an isolated exception; e.g., one individual on Grand Bahama). Consequently, we suspect that many sites not listed that hosted larger numbers of Short-billed Dowitchers might actually cross IBA and WHSRN abundance thresholds for the *griseus* subspecies, and that thresholds we did identify may represent a larger proportion of *griseus* birds than we report.

A similar taxonomic issue exists for the Black-bellied Plover, as some authorities recognize two subspecies in North America (e.g., Brown et al., 2001; Andres et al., 2012) and some do not (Clements et al., 2019). Engelmoer & Roselaar (1998) proposed three subspecies globally as they found that across breeding populations wing, tarsi, and bill length, as well as body mass, varies geographically.

### Existing shorebird survey data are incomplete

The eBird database serves as a central repository for multiple formal shorebird surveys as well as casual observations and is an invaluable tool for exploring bird abundances across regions. However, not all shorebird data collected are entered into eBird. There was no support in our analysis for the recently designated WHSRN site of Regional Importance Cargill Salt Ponds in Bonaire to be deemed an important site for shorebirds or for any particular species although it supports more than 20,000 shorebirds annually and more than 1% of the population of Short-billed Dowitcher (*Limnodromus griseus griseus/hendersoni*) and *rufa* Red Knot (whsrn.org/whsrn_sites/cargill-salt-ponds-bonaire/; accessed 18 May 2020). The entire salt production facility is privately owned so perhaps it is not open to the public and/or the surveyors for site designation did not use eBird to record their counts. The site is listed as an IBA (AN014), last assessed in 2007, with the trigger species including American Flamingo (*Phoenicopterus ruber*) and various terns (http://datazone.birdlife.org/site/factsheet/pelkermeer-saltworks-bonaire-iba-bonaire-sint-eustatius-and-saba-(to-netherlands)/details; Accessed 20 June 2020). In addition, some other sites recognized as crossing population thresholds in a recent wetland study in Cuba (Aguilar et al., 2019) do not appear in eBird.

It is also possible that there are other sites with high shorebird abundances that are not visited due to inaccessibility or are not visited by those who use eBird. As an anecdote, in the ten-year period covered by our study, Puerto Rico boasted the most checklists overall (22% of the total), and Cuba with just over half of that (13%), even though Cuba is orders of magnitude larger in size and population. It also appeared that the majority of those checklists were recorded in western Cuba, with large areas of eastern Cuba absent of any checklists with shorebird records.

Shorebird hunting—both legal and illegal –persists on many islands in the Caribbean such as Barbados, Guadeloupe, Martinique, Saint Barthélemey, Saint Martin, and Trinidad and Tobago, among others (AFSI Harvest Working Group, 2017). Tens of thousands of birds, including Lesser and Greater Yellowlegs, American Golden-Plover, Black-bellied Plover, Whimbrel, and many sandpiper species, are harvested annually in the region (Andres, 2017; Wege, Burke & Reed, 2014). For example, between 2001–2010 it was estimated that the Lesser Yellowlegs harvest on Barbados varied between 5,700 and 19,990 birds per year (Reed, 2012). These data are not captured in eBird.

Finally, in our analysis we discovered an issue with the way that eBird automatically bins checklists as occurring within an IBA. First, there were a few IBAs seemingly not inputted, therefore they would not register. Second, there were spatial errors when assigning coordinates to some checklists that required manual truthing (Fig. S6).

### Conclusions and recommendations

The insular Caribbean historically has not been a priority region for migratory North American shorebirds, but it should receive greater attention. Several sites may qualify for special designation such as IBA or WHSRN, especially Humedal Sur de Pinar del Río in Cuba, which is already an IBA, and Monte Cristi in Dominican Republic. That these thresholds were exceeded using data from only point-in-time counts, it is likely that these –and other sites –actually support even more birds across the year. The geopolitical nature of the Caribbean makes consistent monitoring difficult, but not unimportant. Funding governments and organizations that prioritize shorebird research and successful monitoring schemes such as the Caribbean Waterbird Census, which accounted for more than one-fifth of all high-count data, is essential to close knowledge gaps. Based on our results, we recommend more extensive and systematic shorebird survey coverage, in particular expanding the Caribbean Waterbird Census coverage. Doing this will require more local training and resources. We think it is also important to assimilate existing shorebird survey data that are not currently in eBird and getting them into that data repository. Research on shorebird turnover rates and movements between sites would improve our assessment of site use by shorebirds. Finally, it is important to clarify Black-necked Stilt, Black-bellied Plover, Snowy Plover, Killdeer, and Short-billed Dowitcher subspecies and geographic population abundances and distributions in the Caribbean.

##  Supplemental Information

10.7717/peerj.9831/supp-1 Supplemental Information 1 Details on high-count records and additional data for Snowy Plover and Black-necked Stilt

10.7717/peerj.9831/supp-2 Supplemental Information 2 Aggregated list of sites known to be or potentially important to shorebirds in the insular Caribbean This list of sites includes those from Table S1, the max counts from unique sites in Tables 1-3, and information on the two Caribbean WHSRN sites.

10.7717/peerj.9831/supp-3 Supplemental Information 3 Caribbean IBAs that were triggered by shorebird species Birdlife International established twenty-one IBAs that were triggered by shorebird species (datazone.birdlife.org). Our analysis identified seven of these IBAs that hosted shorebird records that exceeded population thresholds.

10.7717/peerj.9831/supp-4 Supplemental Information 4 Conservation status of species considered for this study

10.7717/peerj.9831/supp-5 Supplemental Information 5 Abundance by species for Figure 2 Bird abundances at Monte Cristi, Dominican Republic and Humedal Sur de Pinar del Río, Cuba during surveys in November 2013 and January 2014, respectively.

10.7717/peerj.9831/supp-6 Supplemental Information 6 Number of unique eBird checklists with shorebird records in the insular Caribbean, by month (2010-2019) Seasons indicated by background color.

10.7717/peerj.9831/supp-7 Supplemental Information 7 Number of unique eBird checklists with shorebird records in the insular Caribbean, by country (2010-2019)

10.7717/peerj.9831/supp-8 Supplemental Information 8 High-count records (with abundance and location, by season) of 31 species of shorebirds

10.7717/peerj.9831/supp-9 Supplemental Information 9 High-count records by country with corresponding shorebird species diversity Bars indicate number of high-count records per country. Points indicate number of shorebird species included in high-count records.

10.7717/peerj.9831/supp-10 Supplemental Information 10 Protocols associated with the 927 high-count records General eBird checklists (blue) accounted for 77% of the total high-count records and were comprised of six protocol types with “traveling” (darkest blue) as the most common. Caribbean Waterbird Census protocols (green) accounted for 22% and were comprised of three types with “CWC Area Search” (darkest green) as the most common. International Shorebird Survey accounted for 1% and has one protocol type (red).

10.7717/peerj.9831/supp-11 Supplemental Information 11 Examples of eBird checklists identified as within or outside of the boundaries of an IBA (A) An example of a checklist we marked as within an IBA even though the checklist coordinates fell outside the IBA boundary. We considered checklist S9591599 (yellow marker) as occurring within the Trou Caïman IBA in Haiti (red shape). Trou Caïman is a shallow freshwater lake, surrounded by subtropical dry forest. Map data: Google, Maxar Technologies (B) An example of a checklist we marked as not within an IBA even though the checklist coordinates were close to the IBA boundary. We considered checklist S19553475 (yellow marker) as occurring outside the Caroni Swamp IBA in Trinidad and Tobago (red shape). The checklist coordinates fall within the Caroni rice fields, an agricultural area different from the habitat of the IBA and separated by a highway. Map data: Google, Maxar Technologies

## Additional Information and Declarations

### Data Availability

The following information was supplied regarding data availability: The data is available at Figshare: Cañizares, Jessica; Reed, J. Michael (2020): Caribbean Shorebirds eBird Raw Data. figshare. Dataset. https://doi.org/10.6084/m9.figshare.12760202.v1.

## References

[ref-1] Atlantic Flyway Shorebird Initiative (2015). Atlantic Flyway Shorebird Initiative Business Plan. https://atlanticflywayshorebirds.org/documents/AFSI_Business_Plan_2015.pdf.

[ref-2] AFSI Harvest Working Group (2017). Achieving a sustainable shorebird harvest in the Caribbean and northern South America. Unpublished report, U.S. Fish and Wildlife Service.

[ref-3] Aguilar S, Manica LT, Acosta M, Castro R, Hernández Z, González A, López M, Mugica L (2019). Spatio-temporal patterns of waterbird assemblages in Cuba’s south coast wetlands: conservation implications. Wetlands.

[ref-4] Andres BA (2017). Current harvest policies and management actions and recent changes for the Caribbean, North America and northern South America, 2012– 2017.

[ref-5] Andres BA, Smith PA, Morrison RIG, Gratto-Trevor CL, Brown SC, Friis CA (2012). Population estimates of North American shorebirds, 2012. Wader Study Group Bulletin.

[ref-6] Bacon PR (1987). Use of wetlands for tourism in the insular Caribbean. Annals of Tourism Research.

[ref-7] Baker AJ, Gonzalez PM, Piersma T, Niles LJ, De Lima Serrano do Nascimento I, Atkinson PW, Clark NA, Minton CDT, Peck MK, Aarts G (2004). Rapid population decline in red knots: fitness consequences of decreased refuelling rates and late arrival in Delaware Bay. Proceedings of the Royal Society B: Biological Sciences.

[ref-8] Bart J, Brown S, Harrington B, Guy Morrison RI (2007). Survey trends of North American shorebirds: population declines or shifting distributions?. Journal of Avian Biology.

[ref-9] Bildstein KL, Bancroft GT, Dugan PJ, Gordon DH, Erwin M, Nol E, Payne LX, Senner SE, Bildstein KL, Bancroftr GT, Dugan PJ, Gordon DH, Erwin RM, Nol E, Payne LX, Senner SE (1991). Approaches to the conservation of coastal wetlands in the western hemisphere. Wilson Bulletin.

[ref-10] Brown AC (2012). Extirpation of the Snowy Plover (*Chardrius alexandrinus*) on St. Martin, West Indies. Journal of Caribbean Ornithology.

[ref-11] Brown SC, Hickey C, Harrington B, Gill R (2001). The U.S. Shorebird Conservation Plan.

[ref-12] Buehler DM, Castillo AI, Angehr G (2002). Shorebird counts in the upper bay of Panama highlight the importance of this key site and the need to improve its protection. Wader Study Group Bulletin.

[ref-13] Buler JJ, Moore FR (2011). Migrant-habitat relationships during stopover along an ecological barrier: extrinsic constraints and conservation implications. Journal of Ornithology.

[ref-14] Callaghan CT, Gawlik DE (2015). Efficacy of eBird data as an aid in conservation planning and monitoring. Journal of Field Ornithology.

[ref-15] Cartwright M (2019). eBird Checklist S58901146.

[ref-16] Centro Nacional de Áreas Protegidas (CNAP) (2013). Plan del Sistema Nacional de Áreas Protegidas 2014–2020.

[ref-17] Clements JF, Schulenberg TS, Iliff MJ, Billerman SM, Fredericks TA, Sullivan BL, Wood CL (2019). The eBird/Clements Checklist of Birds of the World: v2019. https://www.birds.cornell.edu/clementschecklist/download/.

[ref-18] Collazo JA, Harrington BA, Grear JS, Colon JA (1995). Abundance and distribution of shorebirds at the Cabo-Rojo Salt Flats, Puerto-Rico. Journal of Field Ornithology.

[ref-19] Donald PF, Fishpool LDC, Ajagbe A, Bennun LA, Bunting G, Burfield IJ, Butchart SHM, Capellan S, Crosby MJ, Dias MP, Diaz D, Evans MI, Grimmett R, Heath M, Jones VR, Lascelles BG, Merriman JC, O’brien M, Ramírez I, Waliczky Z, Wege DC (2019). Important Bird and Biodiversity Areas (IBAs): the development and characteristics of a global inventory of key sites for biodiversity. Bird Conservation International.

[ref-20] D’Urban Jackson J, Bruford MW, Székely T, DaCosta JM, Sorenson MD, Russo IRM, Maher KH, Cruz-López M, Galindo-Espinosa D, Palacios E, De Sucre-Medrano AE, Cavitt J, Pruner R, Morales AL, Gonzalez O, Burke T, Küpper C (2020). Population differentiation and historical demography of the threatened snowy plover *Charadrius nivosus* (Cassin 1858). Conservation Genetics.

[ref-21] Elliott-Smith E, Bidwell M, Holland AE, Haig SM (2015). Data from the 2011 International Piping Plover Census: U.S. Geological Survey Data Series.

[ref-22] Elliott-Smith E, Haig SM, Ferland CL, Gorman LR (2004). Winter distribution and abundance of snowy plovers in SE North America and the West Indies. Wader Study Group Bulletin.

[ref-23] Elphick CS, Roberts DL, Reed JM (2010). Estimated dates of recent extinctions for North American and Hawaiian birds. Biological Conservation.

[ref-24] Engelmoer M, Roselaar C (1998). Geographical variation in waders.

[ref-25] Escudero G, Navedo JG, Piersma T, De Goeij P, Edelaar P (2012). Foraging conditions ”at the end of the world” in the context of long-distance migration and population declines in red knots. Austral Ecology.

[ref-26] Fischer R (2016). eBird Checklist S32909984.

[ref-27] Funk WC, Mullins TD, Haig SM (2007). Conservation genetics of snowy plovers (*Charadrius alexandrinus*) in the Western Hemisphere: population genetic structure and delineation of subspecies. Conservation Genetics.

[ref-28] Galbraith H, DesRochers DW, Brown S, Reed JM (2014). Predicting vulnerabilities of North American shorebirds to climate change. PLOS ONE.

[ref-29] Gerbracht J, Levesque A (2019). The complete checklist of the birds of the West Indies: v1.1. BirdsCaribbean Checklist Committee. http://www.birdscaribbean.org/caribbean-birds/.

[ref-30] Gill F, Donsker D, Rasmussen P (2020). IOC World Bird List (v10.1).

[ref-31] Gorman LR, Haig SM (2002). Distribution and abundance of snowy plovers in eastern Nother America, the Caribbean, and the Bahamas. Journal of Field Ornithology.

[ref-32] Gratto-Trevor C, Haig SM, Miller MP, Mullins TD, Maddock S, Roche E, Moore P (2016). Breeding sites and winter site fidelity of Piping Plovers wintering in The Bahamas, a previously unknown major wintering area. Journal of Field Ornithology.

[ref-33] Haig SM, Oring LW (1985). Distribution and status of the piping plover throughout the annual cycle. Journal of Field Ornithology.

[ref-34] International Wader Study Group (2003). Waders are declining worldwide, Conclusions from the 2003 International Wader Study Group Conference, Cádiz, Spain. Water Study Group Bulletin.

[ref-35] Howe MA, Geissler PH, Harrington BA (1989). Population trends of North American shorebirds based on the International Shorebird Survey. Biological Conservation.

[ref-36] Jackson BJ, Jackson JA Killdeer (*Charadrius vociferus*), version 1.0. In Birds of the World (A. F. Poole and F. B. Gill, Editors). Ithaca: Cornell Lab of Ornithology. https://doi.org/10.2173/bow.killde.01.

[ref-37] Johnson S, Loring P, Jones D, Yates S (2018). Atypical foraging habitat use by Piping Plovers (*Charadrius melodus*) in The Bahamas. Journal of Caribbean Ornithology.

[ref-38] Johnston A, Newson SE, Risely K, Musgrove AJ, Massimino D, Baillie SR, Pearce-Higgins JW (2014). Species traits explain variation in detectability of UK birds. Bird Study.

[ref-39] Jehl Jr JR, Klima J, Harris RE (2020). Short-billed dowitcher (Limnodromus griseus), version 1.0. In Birds of the World (A. F. Poole and F. B. Gill, Editors). In Birds of the World Ithaca: Cornell Lab of Ornithology. https://doi.org/10.2173/bow.shbdow.01.

[ref-40] Juman RA, Ramsewak D (2013). Land cover changes in the Caroni Swamp Ramsar site, Trinidad (1942 and 2007): implications for management. Journal of Coastal Conservation.

[ref-41] Kelling S, Johnston A, Fink D, Ruiz-Gutierrez V, Bonney R, Bonn A, Fernandez M, Hochachka WM, Julliard R, Kraemer R, Guralnick R (2018). Finding the Signal in the Noise of Citizen Science Observations. bioRxiv.

[ref-42] Kirby JS, Stattersfield AJ, Butchart SHM, Evans MI, Grimmett RFA, Jones VR, O’Sullivan J, Tucker GM, Newton I (2008). Key conservation issues for migratory land and waterbird species on the world’s major flyways. Bird Conservation International.

[ref-43] Latta SC (2012). Avian research in the Caribbean: past contributions and current priorities. Journal of Field Ornithology.

[ref-44] Mathot KJ, Smith BD, Elner RW (2007). Latitudinal clines in food distribution correlate with differential migration in the Western Sandpiper. Ecology.

[ref-45] Mawhinney KP, Hicklin PW, Boates JS (1993). A re-evaluation of the numbers of migrant semipalmated sandpipers, *Calidris pusilla*, in the Bay of Fundy during fall migration. Canadian Field-Naturalist.

[ref-46] Mugica L, Costa MAA, Iménez ARJ, Odríguez ANR, Biología FDe, Habana UDLa, Habana CDLa (2012). Current knowledge and conservation of Cuban waterbirds and their habitats. Journal of Caribbean Ornithology.

[ref-47] Murray NJ, Clemens RS, Phinn SR, Possingham HP, Fuller RA (2014). Tracking the rapid loss of tidal wetlands in the Yellow Sea. Frontiers in Ecology and the Environment.

[ref-48] Murray NJ, Fuller RA (2015). Protecting stopover habitat for migratory shorebirds in East Asia. Journal of Ornithology.

[ref-49] Myers JP, Morrison RIG, Antas PZ, Harrington BA, Lovejoy TE, Sallaberry M, Senner SE, Tarak A (1987). Conservation strategy for migratory species. American Scientist.

[ref-50] North American Bird Conservation Initiative (2016). The state of North America’s birds 2016.

[ref-51] Nguyen K (2019). eBird Checklist S62753014.

[ref-52] Niles L, Sitters A, Dey A, Red Knot Status Assessment Group (2010). Red knot conservation plan for the western hemisphere (Calidris canutus), Version 1.1.

[ref-53] Nol E, MacCulloch K, Pollock L, McKinnon L (2014). Foraging ecology and time budgets of non-breeding shorebirds in coastal Cuba. Journal of Tropical Ecology.

[ref-54] Page GW, Stenzel LE, Warriner JS, Warriner JC, Paton PW (2020). Snowy Plover (Charadrius nivosus), version 1.0. In Birds of the World (A. F. Poole, Editor). Ithaca: Cornell Lab of Ornithology. https://doi.org/10.2173/bow.snoplo5.01.

[ref-55] Parks MA, Collazo JA, Ramos-Alvarez KR (2016a). Factors affecting wetland connectivity for wintering semipalmated sandpipers (*Calidris pusilla*) in the Caribbean. Waterbirds.

[ref-56] Parks MA, Collazo JA, Ramos-Alvarez KR (2016b). Numbers and species composition of resident and migratory shorebirds at the Cabo Rojo salt flats, Puerto Rico (1985-2014). Waterbirds.

[ref-57] Pfister C, Harrington BA, Lavine M (1992). The impact of human disturbance on shorebirds at a migration staging area. Biological Conservation.

[ref-58] Raffaele HA, Wiley J, Garrido OH, Raffaele JI, Keith A (2020). Birds of the West Indies.

[ref-59] Ramsar P (2013). The Ramsar Convention Manual: a guide to the Convention on Wetlands (Ramsar, Iran, 1971).

[ref-60] Reed ET (2012). Evaluation of the Barbados shorebird harvest between 1988 and 2010.

[ref-61] Reed JM, Silbernagle MD, Evans K, Engilis Jr A, Oring LW (1998). Subadult movement patterns of the endangered Hawaiian stilt (*Himantopus mexicanus knudseni*). The Auk.

[ref-62] Robinson JA, Reed JM, Skorupa JP, Oring LW (2020). Black-necked Stilt (Himantopus mexicanus), version 1.0. In Birds of the World (A. F. Poole and F. B. Gill, Editors). Ithaca: Cornell Lab of Ornithology.

[ref-63] Rosenberg KV, Dokter AM, Blancher PJ, Sauer JR, Smith AC, Smith PA, Stanton JC, Panjabi A, Helft L, Parr M, Marra PP (2019). Decline of the North American avifauna. Science.

[ref-64] Runge CA, Martin TG, Possingham HP, Willis SG, Fuller RA (2014). Conserving mobile species. Frontiers in Ecology and the Environment.

[ref-65] Runge CA, Watson JEM, Butchart SHM, Hanson JO, Possingham HP, Fuller RA (2015). Protected areas and global conservation of migratory birds. Science.

[ref-66] Scott DA, Carbonell M (1986). A directory of neotropical wetlands.

[ref-67] Sorenson LG, Gerbracht J (2014). Important sites for shorebirds and waterbirds in the Caribbean: report on the first five years (2010–2014) of the Caribbean Waterbird Census (CWC).

[ref-68] Sorenson LG, Haynes-Sutton A, Rivera-Milan F, Gerbracht J (2019). Caribbean waterbird census manual: promoting conservation of waterbirds and wetlands through monitoring.

[ref-69] Strimas-Mackey M, Hochachka WM, Ruiz-Gutierrez V, Robinson OJ, Miller ET, Auer T, Kelling S, Fink D, Johnston A (2020). Best practices for using eBird data. Version 1.0. Ithaca: Cornell Lab of Ornithology. https://cornelllabofornithology.github.io/ebird-best-practices/.

[ref-70] Strimas-Mackey M, Miller E, Hochachka W (2018). https://cornelllabofornithology.github.io/auk/.

[ref-71] Sullivan BL, Wood CL, Iliff MJ, Bonney RE, Fink D, Kelling S (2009). eBird: a citizen-based bird observation network in the biological sciences. Biological Conservation.

[ref-72] Sutherland WJ (1996). Predicting the consequences of habitat loss for migratory populations. Proceedings of the Royal Society B: Biological Sciences.

[ref-73] Szabo JK, Battley PF, Buchanan KL, Rogers DI (2016). What does the future hold for shorebirds in the East Asian –Australasian Flyway?. Emu.

[ref-74] Thomas GH, Lanctot RB, Székely T (2006). Can intrinsic factors explain population declines in North American breeding shorebirds? A comparative analysis. Animal Conservation.

[ref-75] US Shorebird Conservation Plan Partnership (2016). US Shorebirds of Conservation Concern—2016. https://www.shorebirdplan.org/wp-content/uploads/2016/08/Shorebirds-Conservation-Concern-2016.pdf.

[ref-76] US Fish and Wildlife Service (2011). Endangered and threatened wildlife and plants; 90-day finding on a petition to list the snowy plover and reclassify the wintering population of piping plover. Federal Register 76 FR 55638: 55638-55641. https://www.federalregister.gov/documents/2011/09/08/2011-22900/endangered-and-threatened-wildlife-and-plants-90-day-finding-on-a-petition-to-list-the-snowy-plover.

[ref-77] US Fish and Wildlife Service (2019). Abundance and productivity estimates - update: Atlantic Coast piping plover population. Hadley, Massachusetts. https://www.fws.gov/northeast/pipingplover/pdf/Abundance-Productivity-2018-Update_final-with-tables.pdf.

[ref-78] Van es B (2017). eBird Checklist.

[ref-79] Waliczky Z, Fishpool LDC, Butchart SHM, Thomas D, Heath MF, Hazin C, Donald PF, Kowalska A, Dias MP, Allinson TSM (2019). Important Bird and Biodiversity Areas (IBAs): their impact on conservation policy, advocacy and action. Bird Conservation International.

[ref-80] Watts BD, Turrin C (2016). Assessing hunting policies for migratory shorebirds throughout the western hemisphere. Wader Study.

[ref-81] Wege DC, Anadon-Irizarry V (2008). Important bird areas in the Caribbean: key sites for conservation. BirdLife Conservation Series, No. 15.

[ref-82] Wege DC, Burke W, Reed ET (2014). Migratory shorebirds in Barbados: hunting, management and conservation. BirdLife International (in collaboration with the Barbados Wildfowlers Association and Canadian Wildlife Service).

[ref-83] Wetlands International (2020). Waterbird Population Estimates. http://wpe.wetlands.org/.

[ref-84] Wilcove DS, Wikelski M (2008). Going, going, gone: is animal migration disappearing?. PLOS Biology.

[ref-85] Wunderle JM, Waide RB, Fernandez J (2009). Seasonal abundance of shorebirds in the Jobos Bay estuary in southern Puerto Rico. Journal of Field Ornithology.

[ref-86] Zdravkovic MG (2013). Conservation Plan for the Wilson’s Plover (*Charadrius wilsonia*).

