# Supplementary material for: Identification of priority shorebird conservation areas in the Caribbean
Source: PeerJ. 2020 Sep 8;8:e9831. doi: 10.7717/peerj.9831 (PMC7485488; doi:10.7717/peerj.9831)
Supplement: Supplemental Information 3 — Birdlife International established twenty-one IBAs that were triggered by shorebird species (datazone.birdlife.org). Our analysis identified seven of these IBAs that hosted shorebird records that exceeded population thresholds. [file peerj-08-9831-s003.pdf]

Table S1:
Caribbean IBAs that were triggered by shorebird species.
Birdlife International established twenty-one IBAs that were triggered by shorebird species (datazone.birdlife.org). Our analysis identified seven of these IBAs that hosted shorebird records that exceeded population thresholds.

Country/ Territory	Site name	IBA code	IBA trigger species (shorebirds only)	Species estimate	Year of estimate	Most recent IBA criteria assessment	Species that exceeded thresholds from our analysis
The Bahamas	Driggs Hill to Mars Bay	BS012	Piping Plover	38	2006	2013	
	Grand Bahama Southern Shore	BS003	Piping Plover	70	2006	2007	
	Joulter Cays	BS041	Piping Plover	326	2012	2012	Piping Plover
	Kemp Cay to Pigeon Cay	BS042	Piping Plover	255	2011	2012	Piping Plover
	South Beach Tidal Flats	BS015	Piping Plover	<50	2006	2007	
	Stafford Creek to Andros Town	BS009	Piping Plover	85	2006	2013	
Barbados	St. Lucy Shooting Swamps	BB002	American Golden-Plover<br>Pectoral Sandpiper<br>Greater Yellowlegs<br>Lesser Yellowlegs<br>*Waterbirds Congregation*	4,050-9,000<br>6,600<br>2,550<br>13,200<br>*20,000*	1991<br>1991<br>1991<br>1991<br>*2007*	2007	
	St. Philip Shooting Swamps	BB006	American Golden-Plover<br>Pectoral Sandpiper<br>Greater Yellowlegs<br>Lesser Yellowlegs<br>*Waterbirds Congregation*	4,050-9,000<br>6,600<br>2,550<br>13,200<br>*20,000*	1991<br>1991<br>1991<br>1991<br>*2007*	2007	
Cuba	Cayería Centro - Oriental de Villa Clara	CU008	Piping Plover	unreported	2004	2008	Black-bellied Plover
	Cayos Romano - Cruz - Megano Grande	CU015	Piping Plover	98	1996-2006	2008	

Table S1:
Caribbean IBAs that were triggered by shorebird species.
Birdlife International established twenty-one IBAs that were triggered by shorebird species (datazone.birdlife.org). Our analysis identified seven of these IBAs that hosted shorebird records that exceeded population thresholds.

**Country/ Territory**	**Site name**	**IBA code**	**IBA trigger species (shorebirds only)**	**Species estimate**	**Year of estimate**	**Most recent IBA criteria assessment**	**Species that exceeded thresholds from our analysis**
	Delta del Cauto	CU020	Least Sandpiper<br>Short-billed Dowitcher<br>Lesser Yellowlegs<br>*Waterbirds Congregation*	10,000<br>3,000<br>5,000<br>*50,000-99,999*	2006<br>2006<br>2006<br>*2006*	2008	
	Gran Humedal del Norte de Ciego de Ávila	CU012	Piping Plover	32	2006	2008	Black-necked Stilt
	Humedal Sur de Pinar del Río	CU003	Short-billed Dowitcher<br>*Waterbirds Congregation*	10,000<br>*50,000-99,999*	2007<br>*2007*	2008	Short-billed Dowitcher; Black-bellied Plover; Wilson's Plover
	Humedal Sur de Sancti Spiritus	CU009	Least Sandpiper<br>Short-billed Dowitcher<br>Lesser Yellowlegs<br>*Waterbirds Congregation*	10,000<br>5,000<br>5,000<br>*100,000-499,999*	2007<br>2007<br>2007<br>*2007*	2008	
Puerto Rico	Jobos Bay	PR013	Wilson's Plover	93	1985-1986	2007	
	Suroeste (includes Cabo Rojo)	PR008	Wilson's Plover<br>Snowy Plover<br>Stilt Sandpiper	294<br>266<br>19,895	1991<br>2004<br>1991	2007	Black-necked Stilt
Trinidad & Tobago	West Coast Mudflats	TT001	Semipalmated Plover<br>Whimbrel<br>Short-billed Dowitcher<br>*Waterbirds Congregation*	1,000-2,000<br>500-1,000<br>750-1,250<br>*20,000-49,999*	2007<br>2007<br>2007<br>*2007*	2007	
	East Caicos and adjacent areas	TC005	Wilson's Plover	2005	30 br. pairs	2007	Piping Plover

Table S1:
Caribbean IBAs that were triggered by shorebird species.
Birdlife International established twenty-one IBAs that were triggered by shorebird species (datazone.birdlife.org). Our analysis identified seven of these IBAs that hosted shorebird records that exceeded population thresholds.

Country/ Territory	Site name	IBA code	IBA trigger species (shorebirds only)	Species estimate	Year of estimate	Most recent IBA criteria assessment	Species that exceeded thresholds from our analysis
Turks and Caicos Islands	Grand Turk Salinas and Shores	TC007	Wilson's Plover<br>Short-billed Dowitcher<br>Lesser Yellowlegs<br>Greater Yellowlegs	30 br. pairs<br>4,000<br>6,000<br>1,000	2005<br>2005<br>2005<br>2005	2007	
	North, Middle and East Caicos Ramsar Site	TC003	Black-bellied Plover<br>Wilson's Plover<br>Least Sandpiper<br>Short-billed Dowitcher<br>Lesser Yellowlegs<br>Greater Yellowlegs<br>*Waterbirds Congregation*	2,500<br>100<br>6,000<br>3,200<br>5,000<br>1,000<br>*20,000*	2005<br>2005<br>2005<br>2005<br>2005<br>2005<br>*2005*	2007	
	Salt Cay Creek and Salinas	TC009	Wilson's Plover	30 br. pairs	2005	2007	
