# Supplementary material for: Identification of priority shorebird conservation areas in the Caribbean
Source: PeerJ. 2020 Sep 8;8:e9831. doi: 10.7717/peerj.9831 (PMC7485488; doi:10.7717/peerj.9831)
Supplement: Supplemental Information 4 [file peerj-08-9831-s004.pdf]

Table S2:
Conservation status of species considered for this study.

**Common Name (Population)**	**Scientific Name**	**IUCN Status**[1]	**USSCP Concern Category**[2]
Black-necked Stilt (N. American)	*Himantopus mexicanus*	LC	Least
Black-necked Stilt (Caribbean)	*H. mexicanus*	LC	
American Avocet	*Recurvirostra americana*	LC	Moderate
American Oystercatcher	*Haematopus palliatus*	LC	Greatest
Black-bellied Plover	*Pluvialis squatarola cynosurae*	LC	Moderate
American Golden-Plover	*P. dominica*	LC	High
Snowy Plover (Gulf Coast and Caribbean)	*Charadrius nivosus nivosus* and *tenuirostris*	NT	Greatest
Wilson's Plover (N. American)	*C. wilsonia wilsonia*	LC	Greatest
Wilson's Plover (Caribbean)	*C. w. wilsonia*	LC	
Wilson's Plover (S. American/Caribbean)	*C. w. cinnamominus*	LC	
Semipalmated Plover	*C. semipalmatus*	LC	Least
Piping Plover (Atlantic)	*C. melodus melodus*	NT	ESA-listed[,3, 4, CE]
Killdeer (N. American)	*C. vociferous vociferus*	LC	Moderate
Killdeer (Caribbean)	*C. v. ternominatus*	LC	
Upland Sandpiper	*Bartramia longicauda*	LC	Least
Whimbrel	*Numenius phaeopus*	LC	High
Hudsonian Godwit	*Limosa haemastica*	LC	High[CT]
Ruddy Turnstone	*Arenaria interpres*	LC	High
Red Knot *(rufa)*	*Calidris canutus rufa*	LC	ESA-listed[3, 4, ,CE]
Stilt Sandpiper	*C. himantopus*	LC	Least
Sanderling	*C. alba*	LC	Moderate
Dunlin	*C. alpine hudsonia*	LC	High
Least Sandpiper	*C. minutilla*	LC	Least
White-rumped Sandpiper	*C. fuscicollis*	LC	Least
Pectoral Sandpiper	*C. melanotos*	LC	High
Semipalmated Sandpiper	*C. pusilla*	LC	High
Western Sandpiper	*C. mauri*	LC	Moderate
Short-billed Dowitcher	*Limnodromus griseus*	LC	High
Wilson's Snipe	*Gallinago delicata*	LC	Least
Wilson's Phalarope	*Phalaropus tricolor*	LC	Least
Spotted Sandpiper	*Actitis macularius*	LC	Least
Solitary Sandpiper	*Tringa solitaria*	LC	Least
Greater Yellowlegs	*T. melanoleuca*	LC	Least
Willet	*T. semipalmata*	LC	High
Lesser Yellowlegs	*T. flavipes*	LC	High

[1] The IUCN Red List of Threatened Species. Version 2019-2. http://www.iucnredlist.org. (Accessed 25 November 2019)

LC = least concern; NT = near threatened. IUCN status is at the species level.

[2] U.S. Shorebird Conservation Plan Partnership. 2016. U.S. Shorebirds of Conservation Concern - 2016. http://www.shorebirdplan.org/science/assessment-conservation-status-shorebirds (Accessed 20 June 2020).

[3] Listed on USA Endangered Species Act (ESA).
U. S. Fish and Wildlife Service. ECOS Environmental Conservation Online System. https://ecos.fws.gov/ecp0/pub/SpeciesReport.do?groups=B&listingType=L&mapstatus=1 (Accessed 18 May 2020).

[4] Listed on Canada's Species at Risk Act (SARA).
Hope D, Pekarik C, Drever M, Smith P, Gratto-Trevor C, Paquet J, Aubry Y, Donaldson G, Friis C, Gurney K, Rausch J, McKellar A, Andres B. 2019. Shorebirds of conservation concern in Canada – 2019. Wader Study. 126:2. 10.18194/ws.00148.

[CE, CT] Listed by the Committee on the Status of Endangered Wildlife in Canada (COSEWIC)
CE= COSEWIC Endangered; CT = COSEWIC Threatened.
Government of Canada. 2020. Species at risk public registry. https://www.canada.ca/en/environment-climate-change/services/species-risk-public-registry.html (Accessed 18 May 2020).
