# Supplementary material for: Identification of priority shorebird conservation areas in the Caribbean
Source: PeerJ. 2020 Sep 8;8:e9831. doi: 10.7717/peerj.9831 (PMC7485488; doi:10.7717/peerj.9831)
Supplement: Supplemental Information 5 — Bird abundances at Monte Cristi, Dominican Republic and Humedal Sur de Pinar del Río, Cuba during surveys in November 2013 and January 2014, respectively. [file peerj-08-9831-s005.pdf]

Table S3:
Abundance by species for Fig. 2 of main text.
Bird abundances at Monte Cristi, Dominican Republic and Humedal Sur de Pinar del Río, Cuba during surveys in November 2013 and January 2014, respectively.

Common Name	Group	Monte Cristi	Humedal Sur de Pinar Del Río
Greater Yellowlegs	Tringa	860	142
Lesser Yellowlegs	Tringa	3219	395
Willet	Tringa	8	117
Wilson's Plover	Charadrius	14	44
Killdeer	Charadrius	14	41
Snowy Plover	Charadrius	0	33
Semipalmated Plover	Charadrius	41	24
Least Sandpiper	Calidrids, Arenaria, Actitius	1859	1550
Semipalmated Sandpiper	Calidrids, Arenaria, Actitius	3139	24
Western Sandpiper	Calidrids, Arenaria, Actitius	154	106
Sanderling	Calidrids, Arenaria, Actitius	20	3
Stilt Sandpiper	Calidrids, Arenaria, Actitius	2213	0
Ruddy Turnstone	Calidrids, Arenaria, Actitius	27	52
Spotted Sandpiper	Calidrids, Arenaria, Actitius	4	3
Black-bellied Plover	Pluvialis	92	132
American Golden-Plover	Pluvialis	0	3
Black-necked Stilt	Himantopus, Recurvirostra, Haematopus	5480	360
American Avocet	Himantopus, Recurvirostra, Haematopus	0	8
American Oystercatcher	Himantopus, Recurvirostra, Haematopus	2	0
Long-billed Dowitcher	Limnodromus	0	70
Short-billed Dowitcher	Limnodromus	3	14517
Whimbrel	Numenius	1	0
Wilson's Snipe	Gallinago	1	6
