# Supplementary figures and images for: Identification of priority shorebird conservation areas in the Caribbean
Source: PeerJ. 2020 Sep 8;8:e9831. doi: 10.7717/peerj.9831 (PMC7485488; doi:10.7717/peerj.9831)

Number of Checklists

30,000

20,000

10,000

Winter

Spring

Summer

Fall

Dec

Jan

Feb

Mar

Apr

May

Jun

Jul

Aug

Sep

Oct

Nov

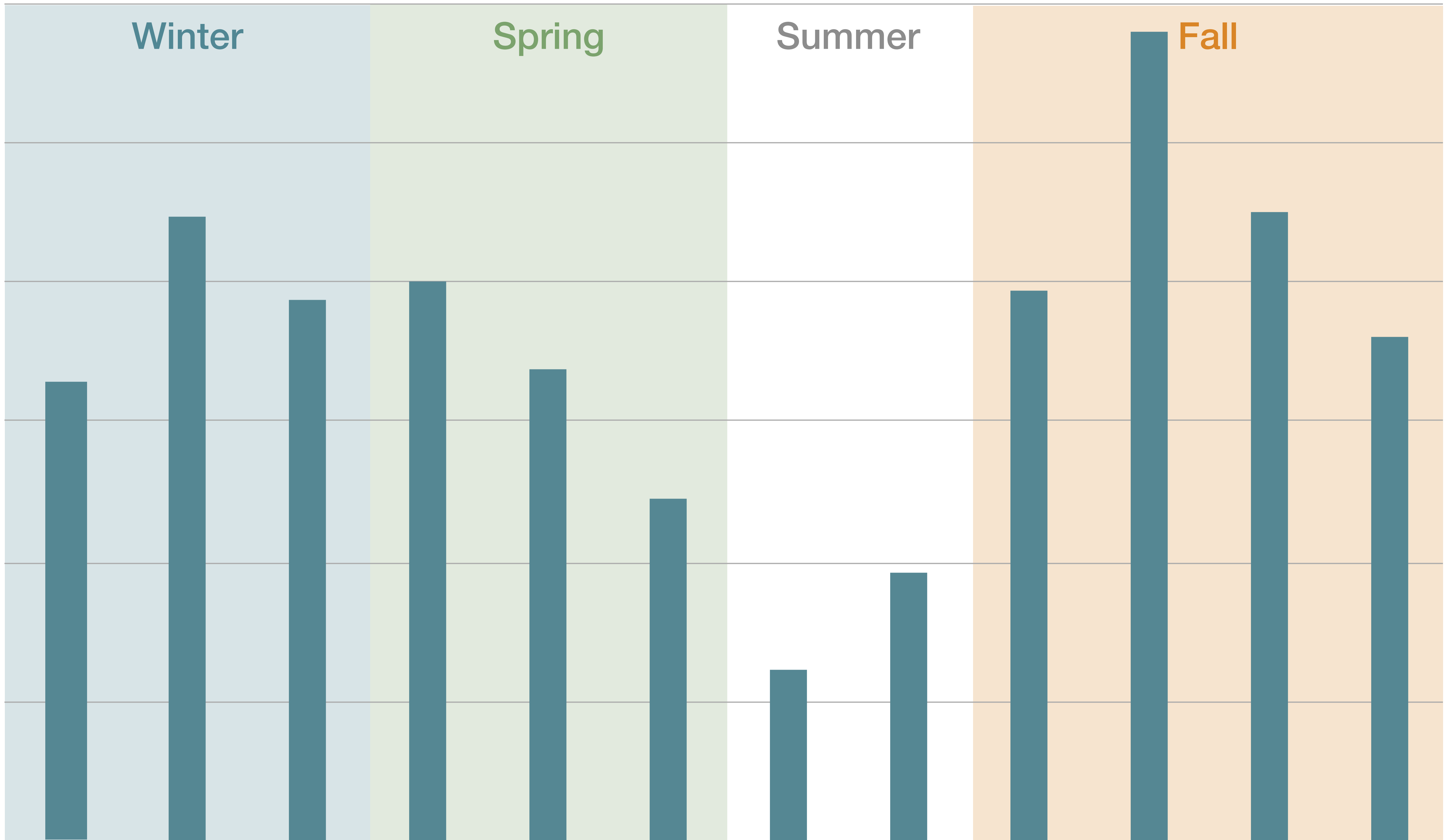

Supplement: Supplemental Information 6 — Seasons indicated by background color. [file peerj-08-9831-s006.pdf]

Number of Checklists

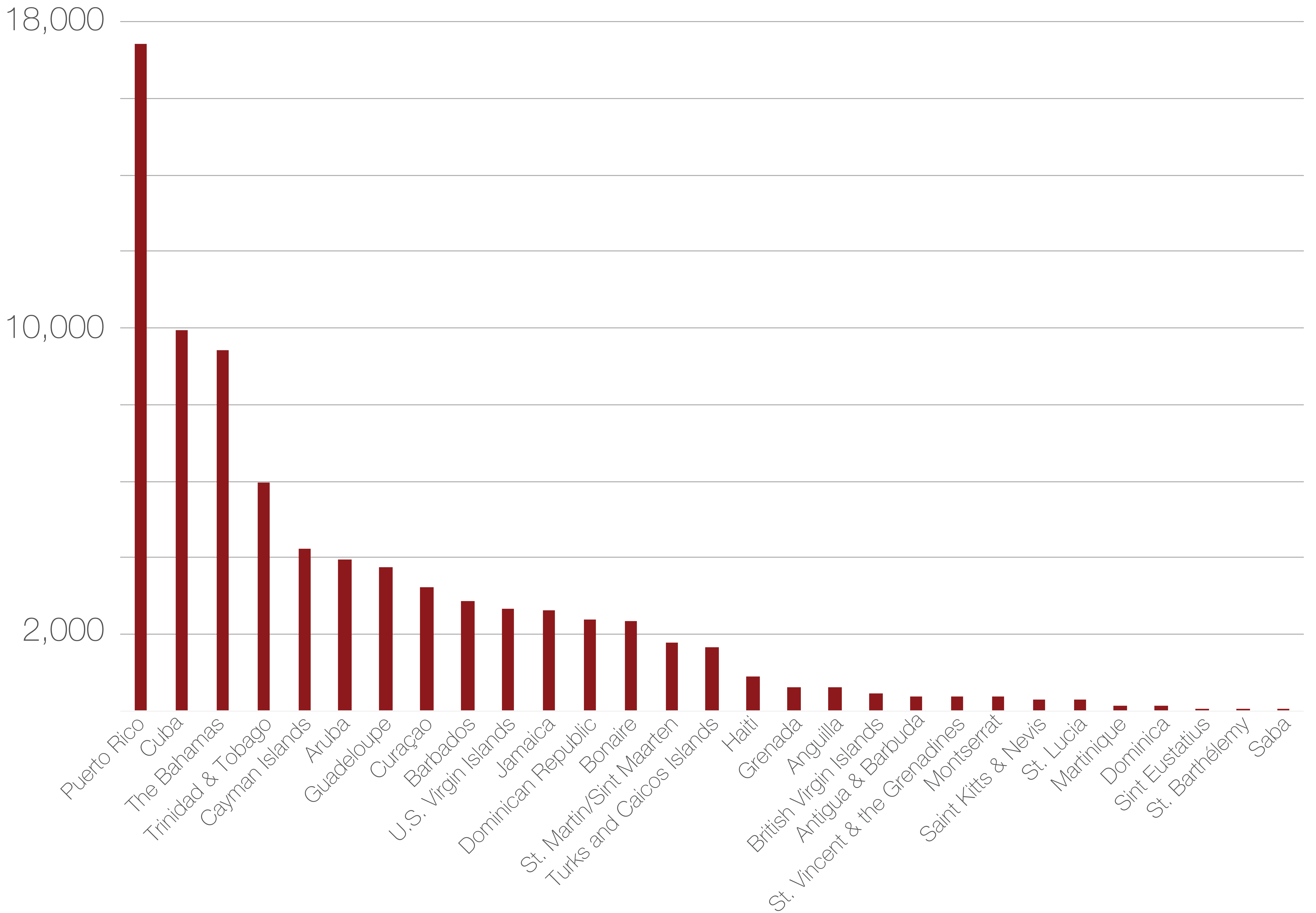

Supplement: Supplemental Information 7 [file peerj-08-9831-s007.pdf]

# Highcount totals per country and corresponding species diversity

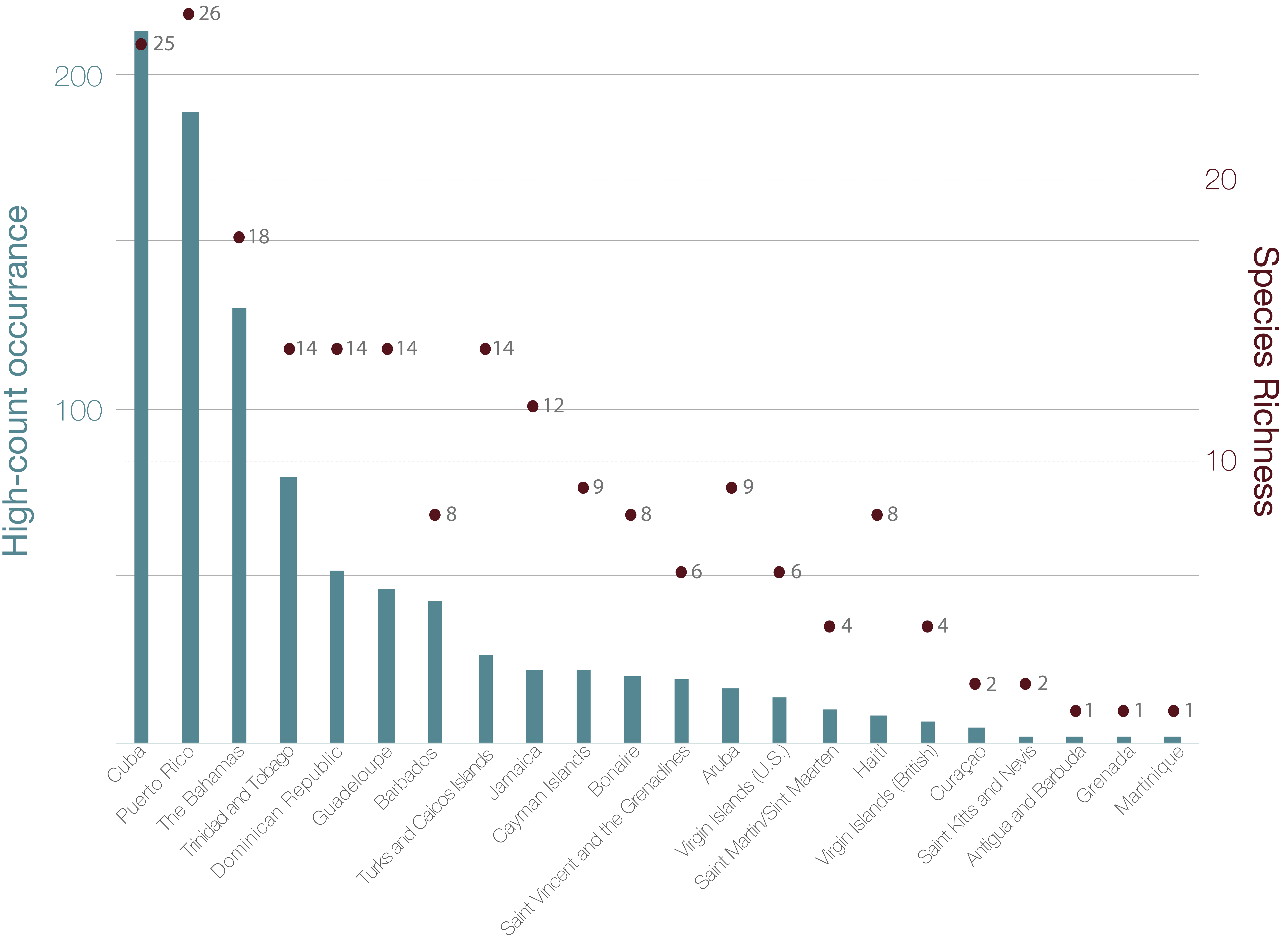

Supplement: Supplemental Information 9 — Bars indicate number of high-count records per country. Points indicate number of shorebird species included in high-count records. [file peerj-08-9831-s009.pdf]

High-count Records

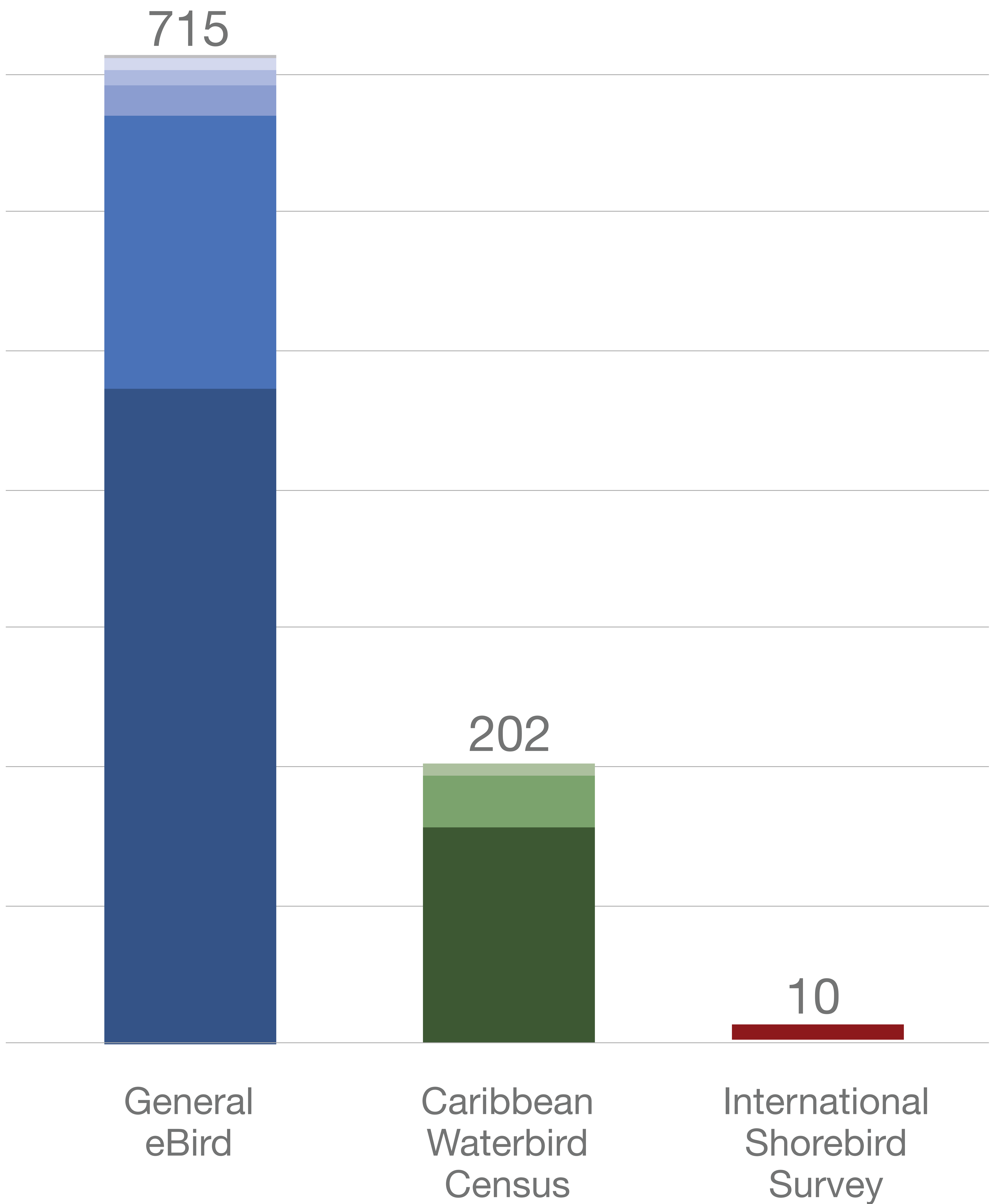

Supplement: Supplemental Information 10 — General eBird checklists (blue) accounted for 77% of the total high-count records and were comprised of six protocol types with “traveling” (darkest blue) as the most common. Caribbean Waterbird Census protocols (green) accounted for 22% and were comprised of three types with “CWC Area Search” (darkest green) as the most common. International Shorebird Survey accounted for 1% and has one protocol type (red). [file peerj-08-9831-s010.pdf]

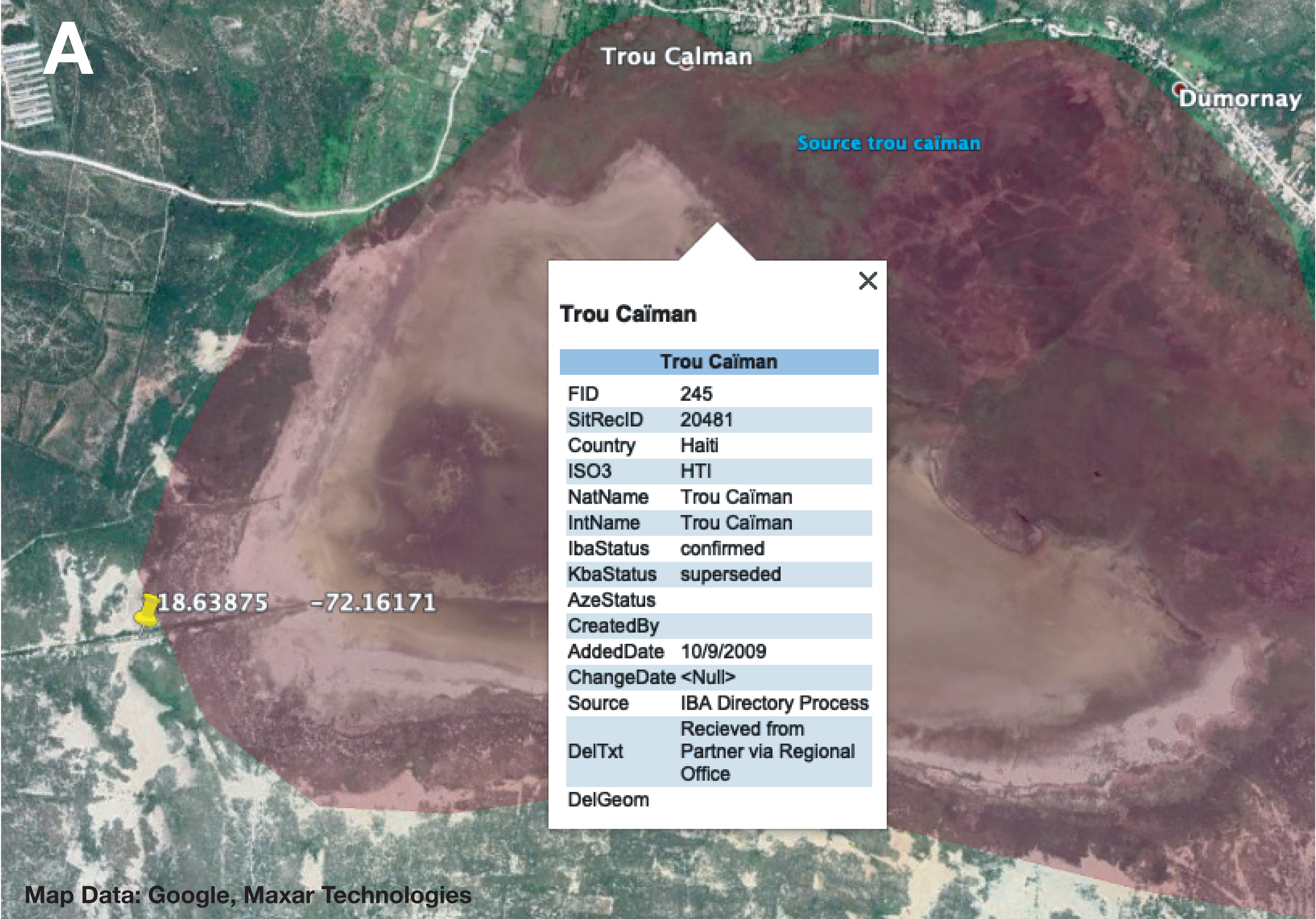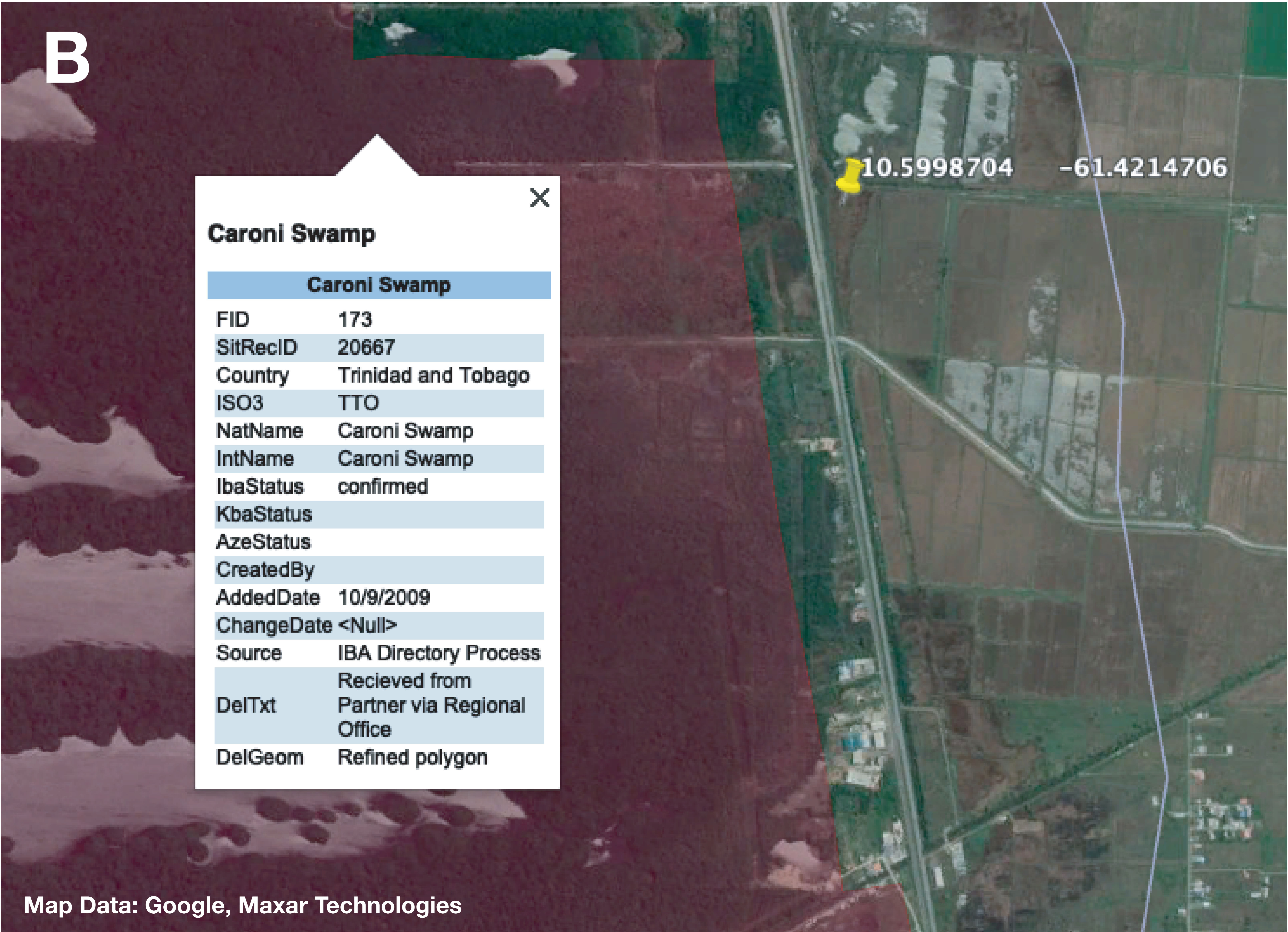

Supplement: Supplemental Information 11 — (A) An example of a checklist we marked as within an IBA even though the checklist coordinates fell outside the IBA boundary. We considered checklist S9591599 (yellow marker) as occurring within the Trou Caïman IBA in Haiti (red shape). Trou Caïman is a shallow freshwater lake, surrounded by subtropical dry forest. Map data: Google, Maxar Technologies (B) An example of a checklist we marked as not within an IBA even though the checklist coordinates were close to the IBA boundary. We considered checklist S19553475 (yellow marker) as occurring outside the Caroni Swamp IBA in Trinidad and Tobago (red shape). The checklist coordinates fall within the Caroni rice fields, an agricultural area different from the habitat of the IBA and separated by a highway. Map data: Google, Maxar Technologies [file peerj-08-9831-s011.pdf]
