# Supplementary material for: Identification of priority shorebird conservation areas in the Caribbean
Source: PeerJ. 2020 Sep 8;8:e9831. doi: 10.7717/peerj.9831 (PMC7485488; doi:10.7717/peerj.9831)
Supplement: Supplemental Information 8 [file peerj-08-9831-s008.pdf]

Supplemental Figure S3

(7 pages, 31 species)

Methods

repeated from subsection “High Counts for Species”

To determine important sites for each species, we identified the ten sites in the Caribbean with the highest counts of each species within each of three seasons. We defined seasons as Fall (August-November), Winter (December-February), and Spring (March-May), to coincide with primarily migratory and non-migratory time periods. We excluded summer months because migrants were largely absent. However, we did scan the summer months and included high counts in July as occurring in the Fall as such an occurrence would coincide with the biology of migration, rather than relying on the artificiality of the calendar; this resulted in one exception, a group of 2350 Lesser Yellowlegs observed on 31 July 2017. In cases where a single large site is comprised of a complex of wetlands that cannot be surveyed in a single visit, counts at unique sites across a three-day period were aggregated to derive the high count for that site (Sorenson & Gerbracht, 2014); the only site where this applied was Monte Cristi, Dominican Republic. However, there is always the chance that birds move between unique sites at wetland complexes; therefore, all counts from unique sites for multi-day data are provided in Data S1 and both the aggregated high count and the single highest count of all unique sites are reported when assessing population thresholds. High-counts of a single individual were excluded. Sites did not repeat within the same season for the same year. That is, if the same wetland had the top 3 (for example) highest counts of a species, but all occurred in the Fall of 2010, we included the site only once on our list with the highest count of the three. If that same wetland had 3 of the highest counts but each in a different year, we included the site three times. In the event that there was a tie in abundance for the 10th place, we included all sites until the tie ceased. In addition, for each high count, we noted whether the checklist had an associated protocol type (i.e., CWC, ISS). Finally, we determined the current IBA status of sites where high counts were recorded by comparing the coordinates of those sites as entered on eBird with a shapefile of IBAs available from BirdLife International (datazone.birdlife.org/site/requestgis).

Seasons

Fall	August - November
Winter	December - February
Spring	March - May

Country/Nation Codes

AB	Antigua and Barbuda	Gr	Grenada
Ar	Aruba	Ha	Haiti
Bb	Barbados	Ja	Jamaica
Bh	Bahamas	Mq	Martinique
Bo	Bonaire	PR	Puerto Rico
BVI	British Virgin Islands	SKN	Saint Kitts & Nevis
Cc	Curaçao	SM	Saint Martin/Sint Maarten
CI	Cayman Islands	SVG	Saint Vincent & the Grenadines
Cu	Cuba	TCI	Turks & Caicos Islands
DR	Dominican Republic	TT	Trinidad & Tobago
Gd	Guadeloupe	USVI	U.S. Virgin Islands

High Counts by Season

Countries of High Counts by Season

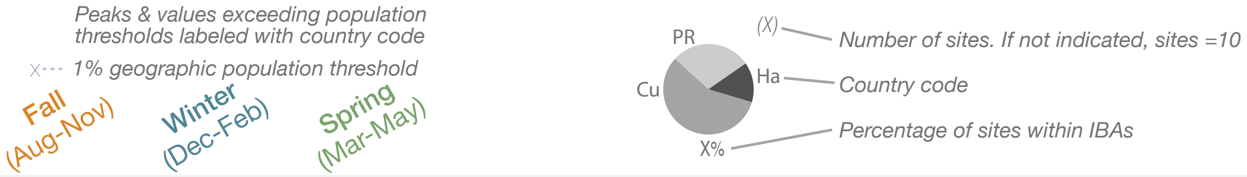

Black-necked Stilt *Himantopus mexicanus*

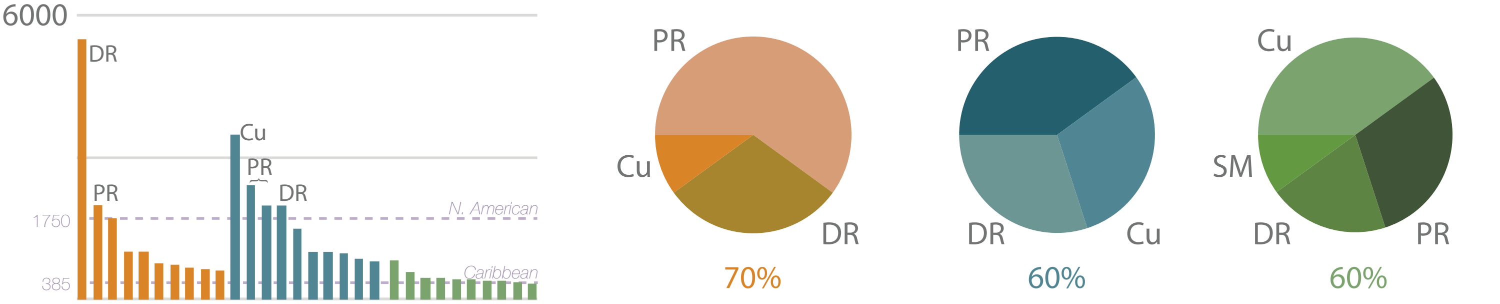

American Avocet *Recurvirostra americana*

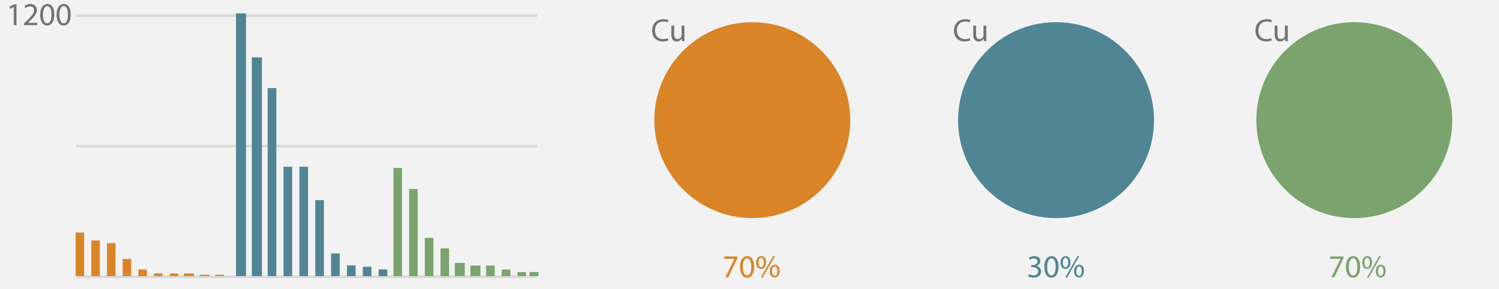

American Oystercatcher *Haematopus palliatus*

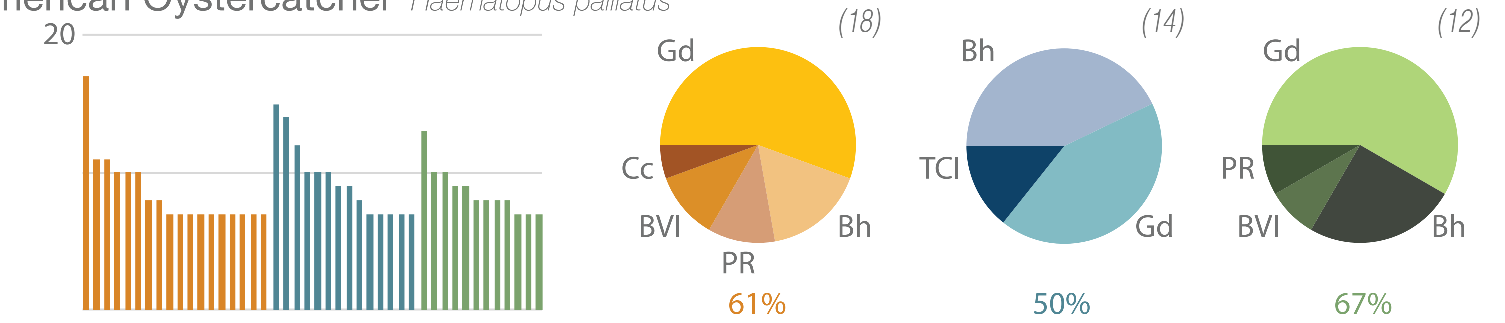

Black-bellied Plover *Pluvialis squatarola*

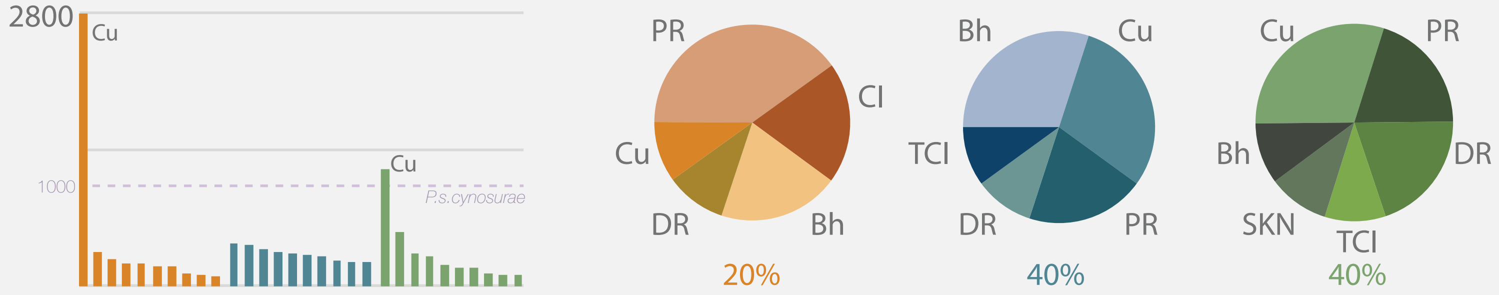

American Golden-Plover *Pluvialis dominica*

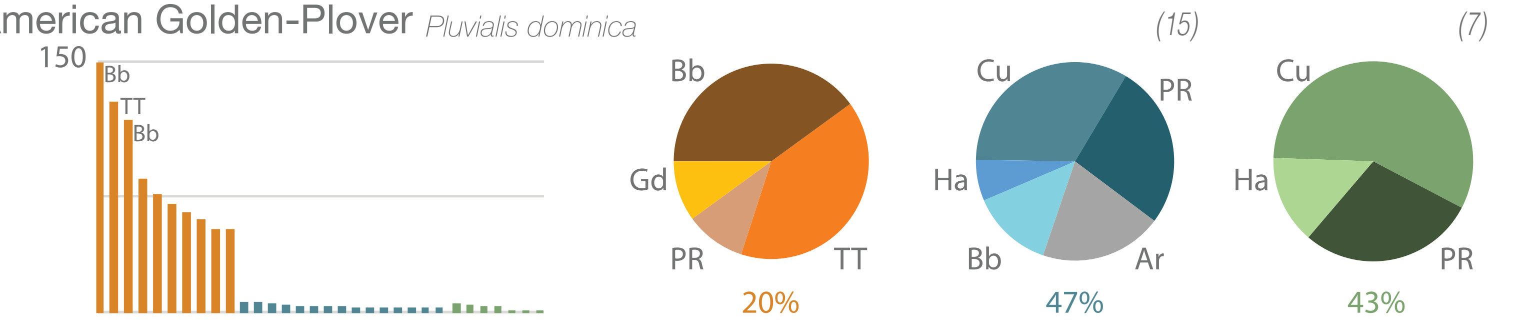

High Counts by Season

Peaks & values exceeding population thresholds labeled with country code

1% geographic population threshold

Fall (Aug-Nov) | Winter (Dec-Feb) | Spring (Mar-May)

Countries of High Counts by Season

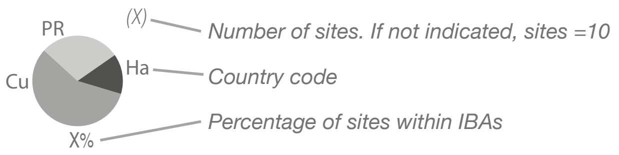

Snowy Plover *Charadrius nivosus*

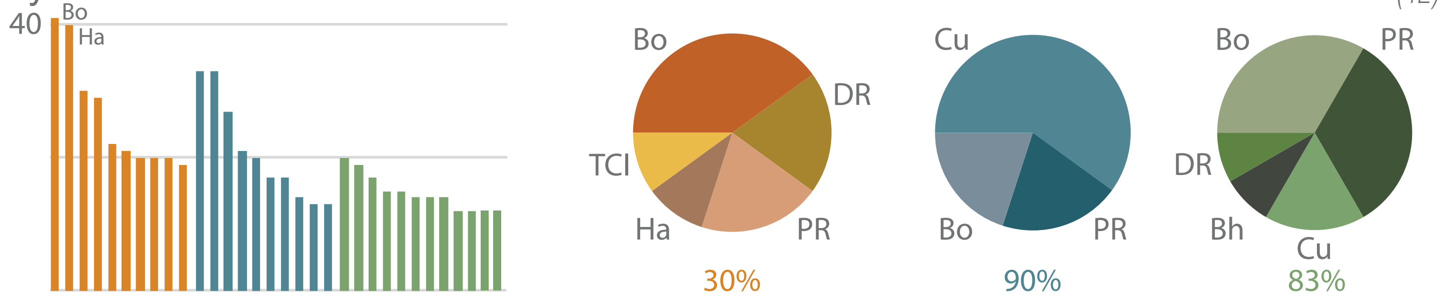

Wilson's Plover *Charadrius wilsonia*

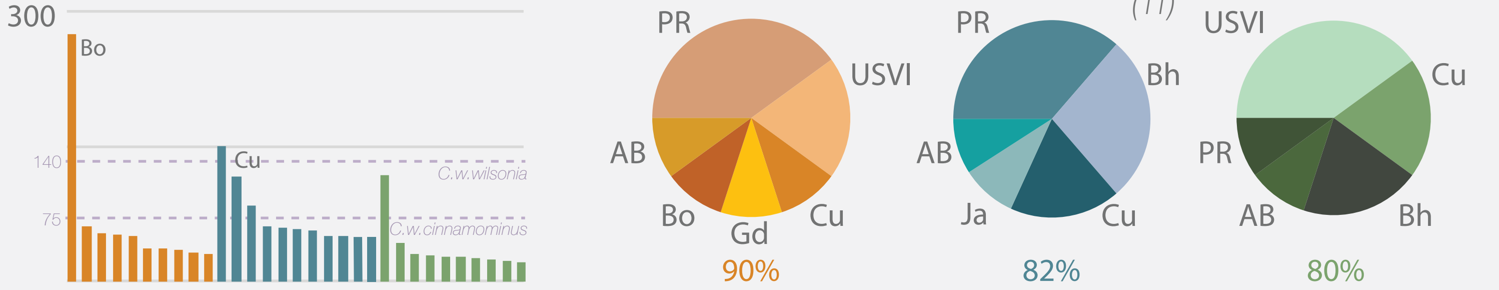

Semipalmated Plover *Charadrius semipalmatus*

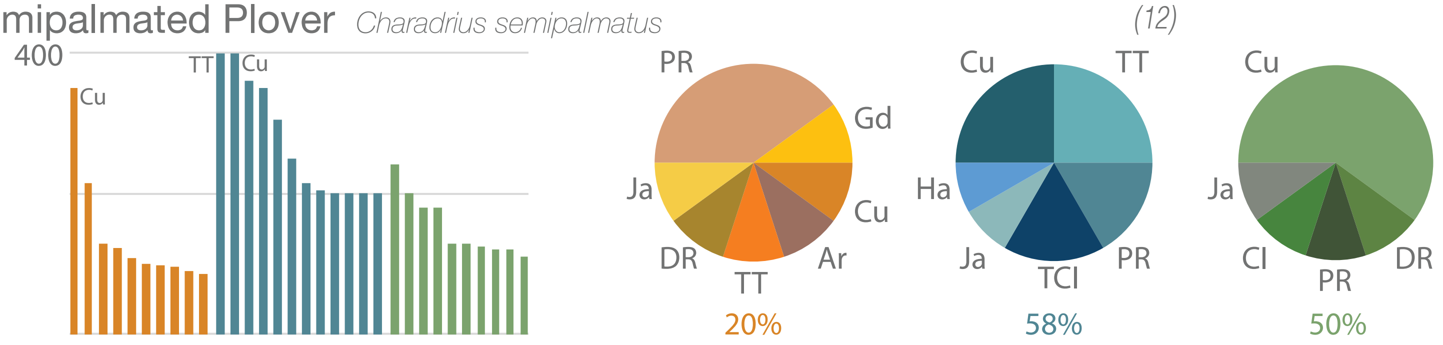

Piping Plover *Charadrius melodus melodus*

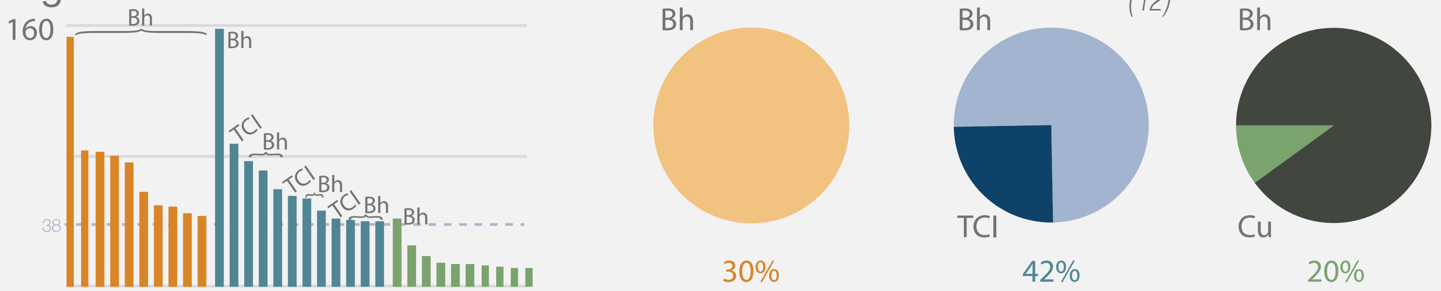

Killdeer *Charadrius vociferus*

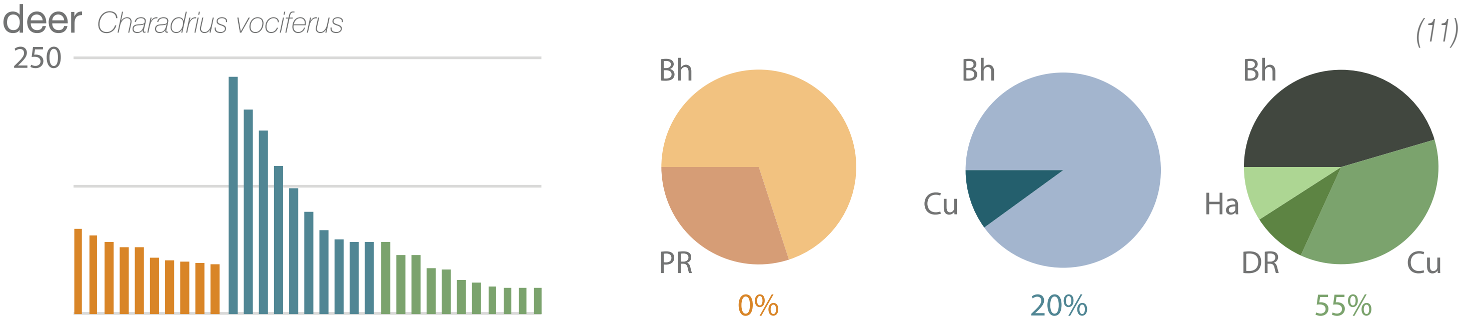

High Counts by Season

Peaks & values exceeding population thresholds labeled with country code

1% geographic population threshold

Fall (Aug-Nov) Winter (Dec-Feb) Spring (Mar-May)

Countries of High Counts by Season

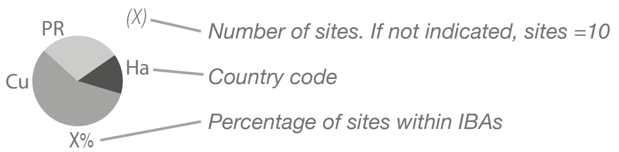

Ruddy Turnstone *Arenaria interpres*

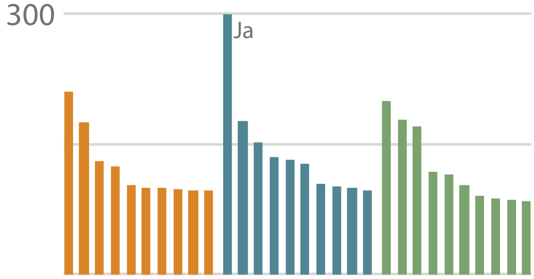

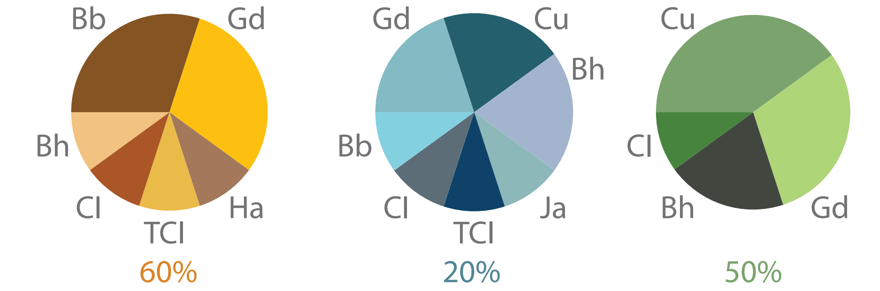

Red Knot *Calidris canutus rufa*

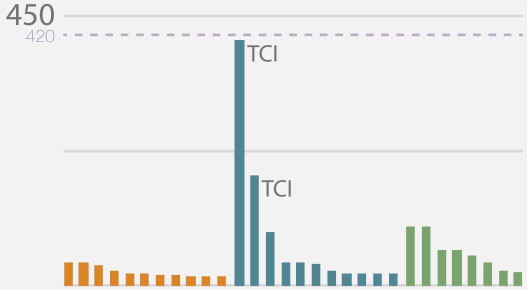

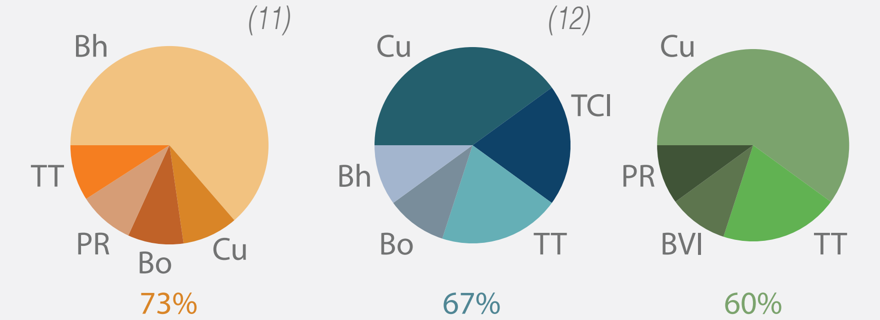

Stilt Sandpiper *Calidris himantopus*

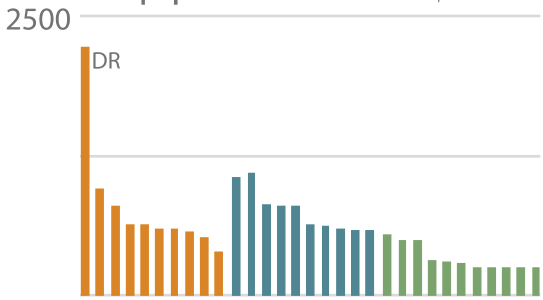

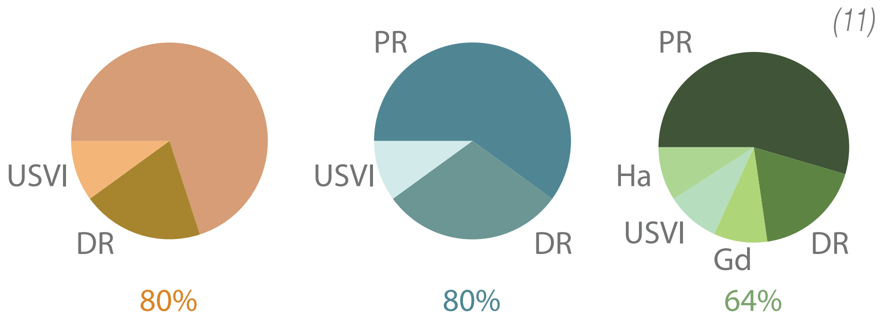

Sanderling *Calidris alba*

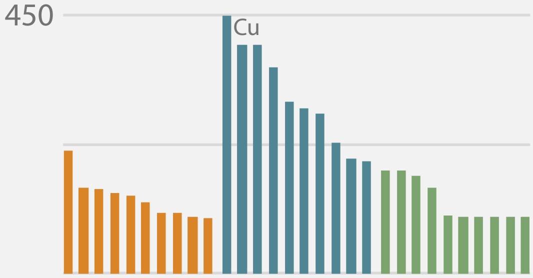

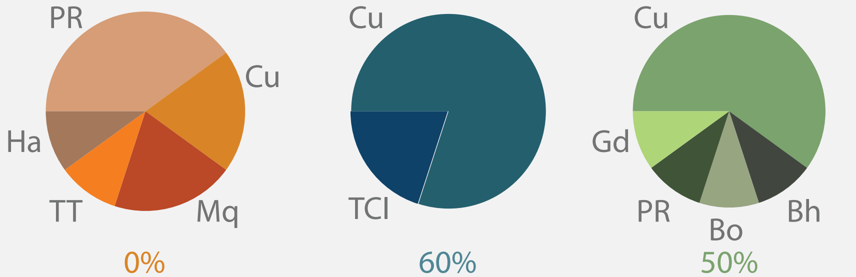

Dunlin *Calidris alpina hudsonia*

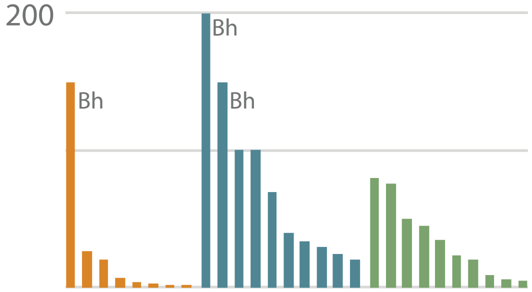

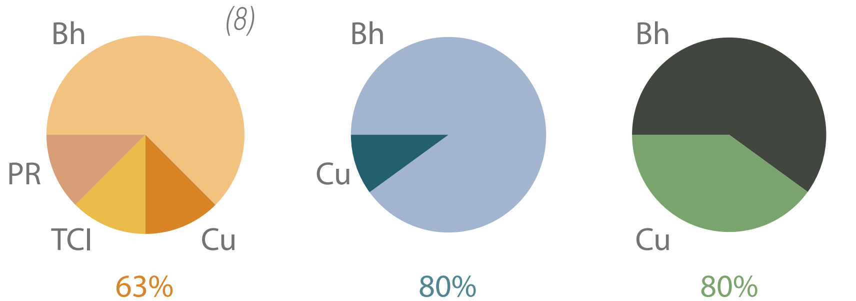

High Counts by Season

Peaks labeled with country code

Fall (Aug-Nov) · Winter (Dec-Feb) · Spring (Mar-May)

Countries of High Counts by Season

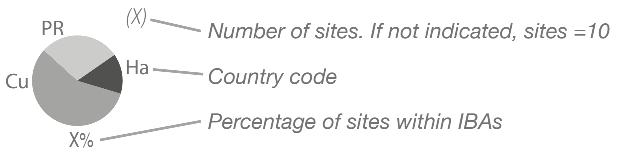

Least Sandpiper *Calidris minutilla*

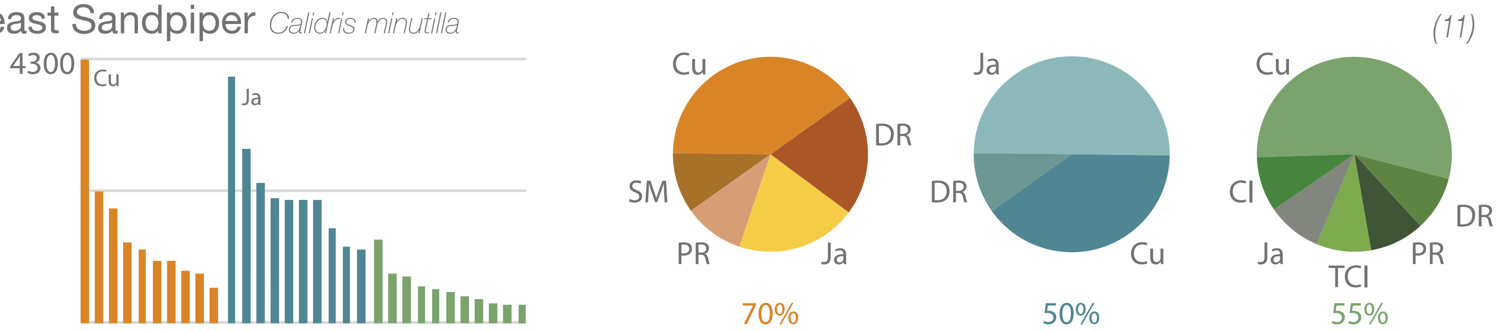

White-rumped Sandpiper *Calidris fuscicollis*

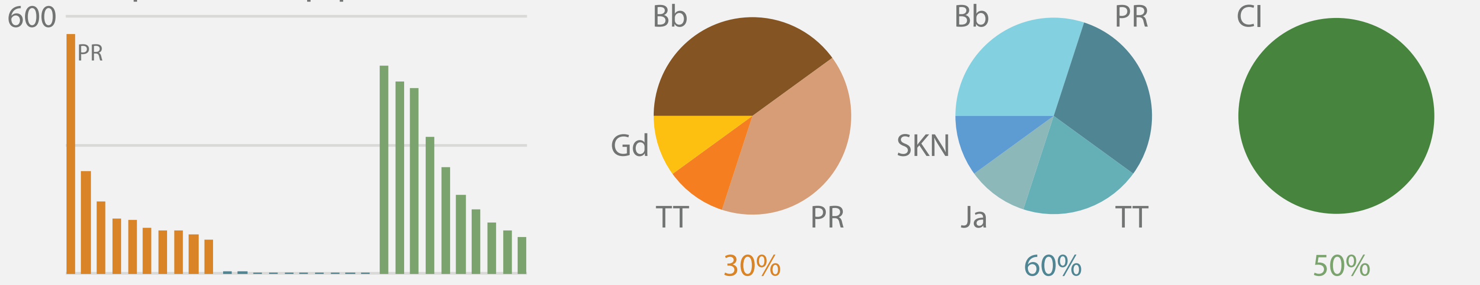

Pectoral Sandpiper *Calidris melanotos*

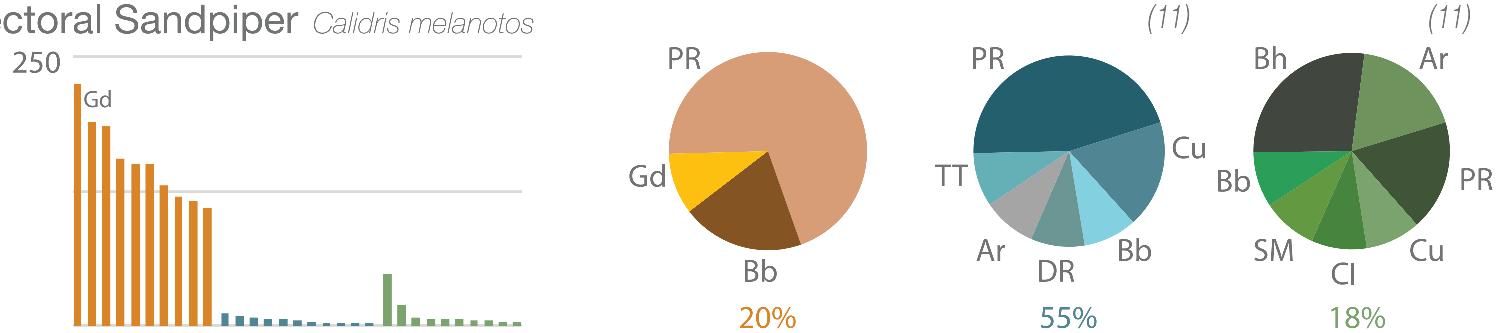

Semipalmated Sandpiper *Calidris pusilla*

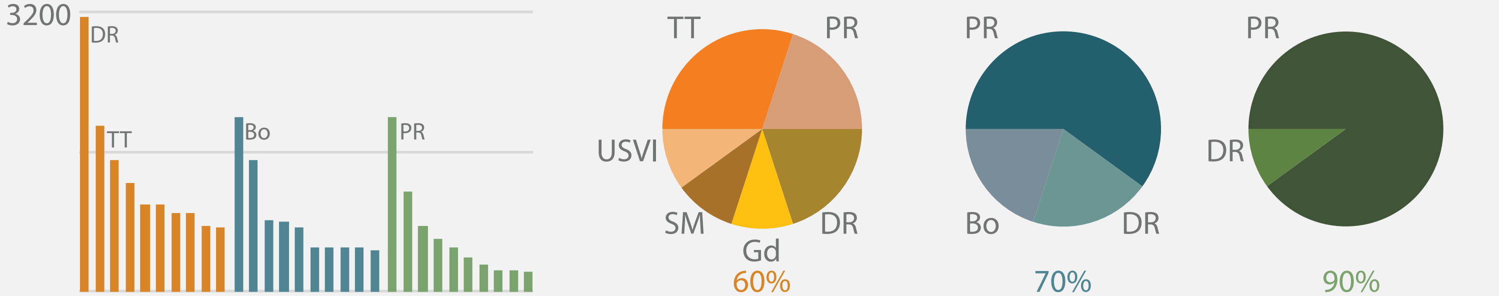

Western Sandpiper *Calidris mauri*

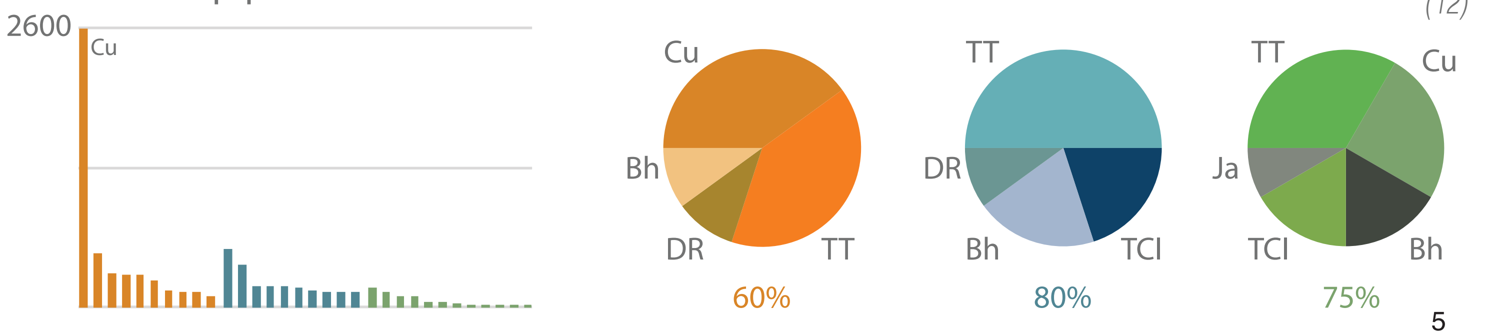

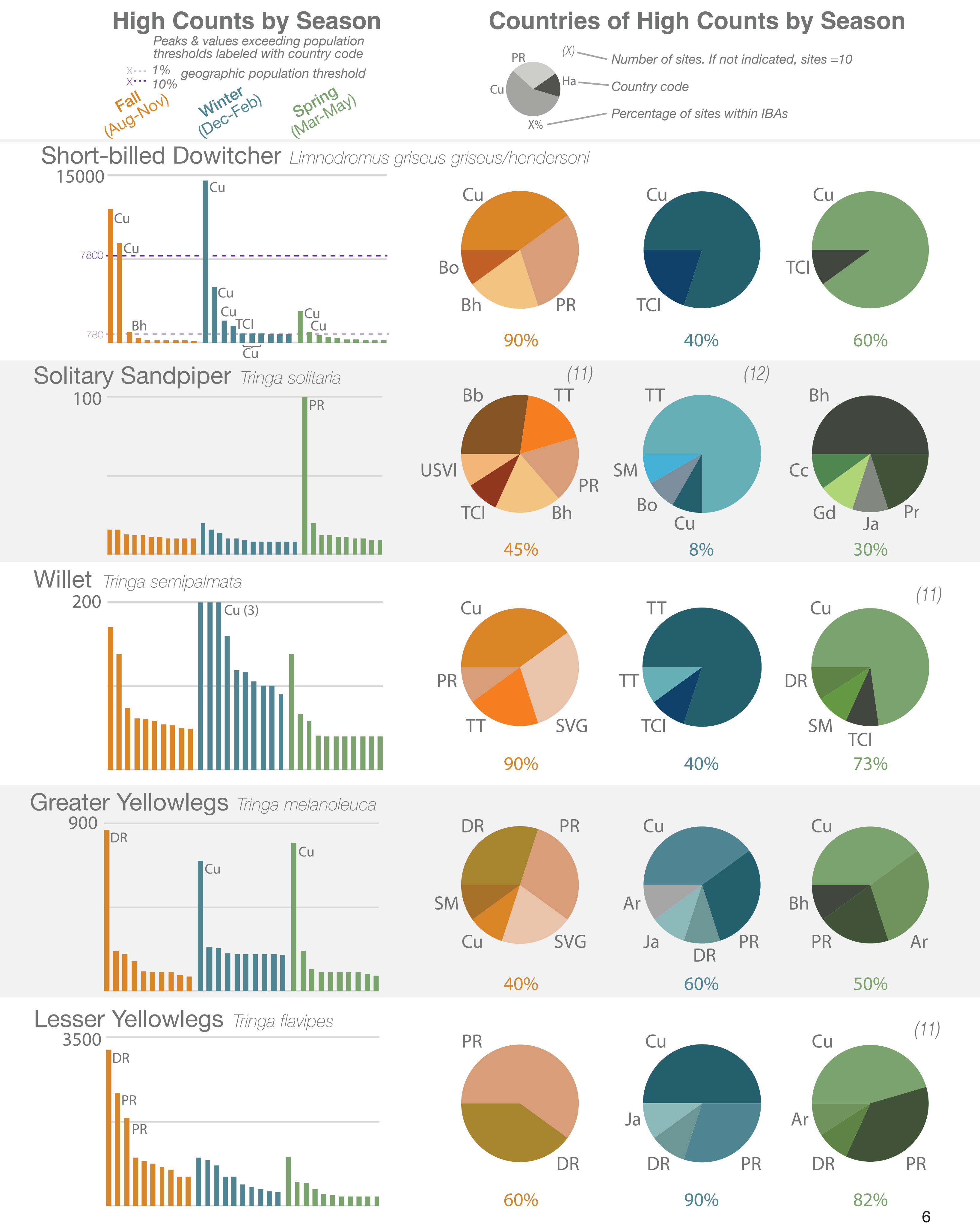
High Counts by Season
Peaks & values exceeding population thresholds labeled with country code
1%
10%
geographic population threshold
Fall (Aug-Nov)
Winter (Dec-Feb)
Spring (Mar-May)
Countries of High Counts by Season
PR
(X)
Number of sites. If not indicated, sites =10
Ha
Country code
Cu
X%
Percentage of sites within IBAs
Short-billed Dowitcher Limnodromus griseus griseus/hendersoni
15000
7800
780
Cu
Cu
Bh
Cu
Cu
Cu
TCI
Cu
Cu
Cu
Bo
Bh
PR
90%
TCI
40%
TCI
60%
Solitary Sandpiper Tringa solitaria
100
PR
(11)
Bb
TT
USVI
PR
TCI
Bh
45%
(12)
TT
SM
Bo
Cu
8%
Bh
Cc
Gd
Ja
Pr
30%
Willet Tringa semipalmata
200
Cu (3)
Cu
PR
TT
SVG
90%
TT
TT
TCI
40%
(11)
Cu
DR
SM
TCI
73%
Greater Yellowlegs Tringa melanoleuca
900
DR
Cu
Cu
DR
PR
SM
Cu
SVG
40%
Cu
Ar
Ja
DR
PR
60%
Cu
Bh
PR
Ar
50%
Lesser Yellowlegs Tringa flavipes
3500
DR
PR
PR
PR
DR
60%
Cu
Ja
DR
PR
90%
(11)
Cu
Ar
DR
PR
82%

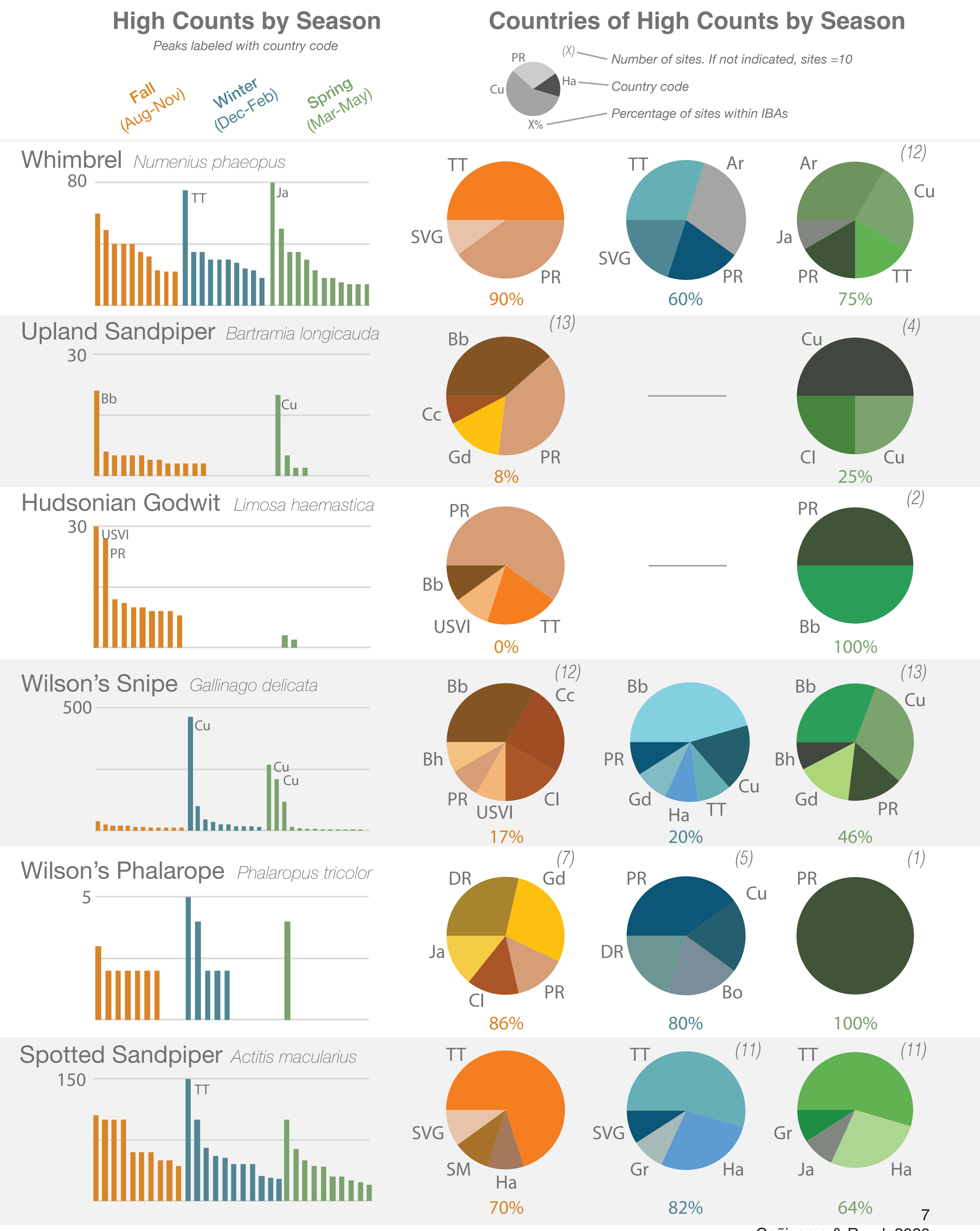
High Counts by Season
Peaks labeled with country code
Fall (Aug-Nov)
Winter (Dec-Feb)
Spring (Mar-May)
Countries of High Counts by Season
PR
(X)
Number of sites. If not indicated, sites =10
Cu
Ha
Country code
X%
Percentage of sites within IBAs
Whimbrel Numenius phaeopus
80
TT
Ja
TT
SVG
PR
90%
TT
Ar
SVG
PR
60%
(12)
Ar
Cu
Ja
PR
TT
75%
Upland Sandpiper Bartramia longicauda
30
Bb
Cu
(13)
Bb
Cc
Gd
PR
8%
(4)
Cu
CI
Cu
25%
Hudsonian Godwit Limosa haemastica
30
USVI
PR
PR
Bb
USVI
TT
0%
(2)
PR
Bb
100%
Wilson's Snipe Gallinago delicata
500
Cu
Cu
Cu
(12)
Bb
Cc
Bh
PR
USVI
CI
17%
Bb
PR
Gd
Ha
TT
Cu
20%
(13)
Bb
Cu
Bh
Gd
PR
46%
Wilson's Phalarope Phalaropus tricolor
5
(7)
DR
Gd
Ja
CI
PR
86%
(5)
PR
Cu
DR
Bo
80%
(1)
PR
100%
Spotted Sandpiper Actitis macularius
150
TT
TT
SVG
SM
Ha
70%
TT
(11)
SVG
Gr
Ha
82%
TT
(11)
Gr
Ja
Ha
64%
